# Supplementary material for: Design and Synthesis of Benzimidazole-Chalcone Derivatives as Potential Anticancer Agents
Source: Molecules. 2019 Sep 6;24(18):3259. doi: 10.3390/molecules24183259 (PMC6767017; doi:10.3390/molecules24183259)
Supplement: Supplementary file 1 [file molecules-24-03259-s001.pdf]

Supporting Information

Design and Synthesis of Benzimidazole-Chalcone Derivatives as Potential Anticancer Agents

Cheng-Ying Hsieh [1], Pi-Wen Ko [2], Yu-Jui Chang [1], Mohit Kapoor [3], Yu-Chuan Liang [4], Hsueh-Liang Chu [5], Hui-Hsien Lin [6], Jia-Cherng Horng [1,*] and Ming-Hua Hsu [7,*]

[1] Department of Chemistry, National Tsing Hua University, Hsinchu 30013, Taiwan; futariwhisper@gmail.com (C.-Y.H.); rick5569268@gmail.com (Y.-J.C.)
[2] Department of Biomedical Engineering and Environmental Sciences, National Tsing Hua University, Hsinchu 30013, Taiwan; koko37bebe@hotmail.com (P.-W.K.)
[3] Chitkara University Institute of Engineering and Technology, Chitkara University, Punjab 140 401, India; mohitkapoor.chemistry@gmail.com (M.K.)
[4] Agricultural Biotechnology Research Center, Academia Sinica, Taipei 11529, Taiwan; ycliang@sinica.edu.tw (Y.-C.L.)
[5] Graduate Institute of Translational Medicine, College of Medicine and Technology, Taipei Medical University, Taipei 11031, Taiwan; szxchu@gmail.com (H.-L.C.)
[6] Division of Radiotherapy, Department of Oncology, Taipei Veterans General Hospital, Taipei 11217, Taiwan; twwarcgogo@gmail.com (H.-H.L.)
[7] Department of Chemistry, National Changhua University of Education, Changhua 50007, Taiwan
* Correspondence: jchorng@mx.nthu.edu.tw (J.-C.H.); minghuahsu@cc.ncue.edu.tw (M.-H.H.); Tel.: +886-3-5715131 (ext. 35635) (J.-C.H.); +886-4-7232105(ext. 3547) (M.-H.H.) Fax: +886-4-7211190 (M.-H.H.)
These authors contributed equally to this work.

Contents

(1) Characterization Data:
^{1}H-NMR, ^{13}C-NMR, IR, HPLC

(2) Flow Cytometry Data

(1) Characterization Data

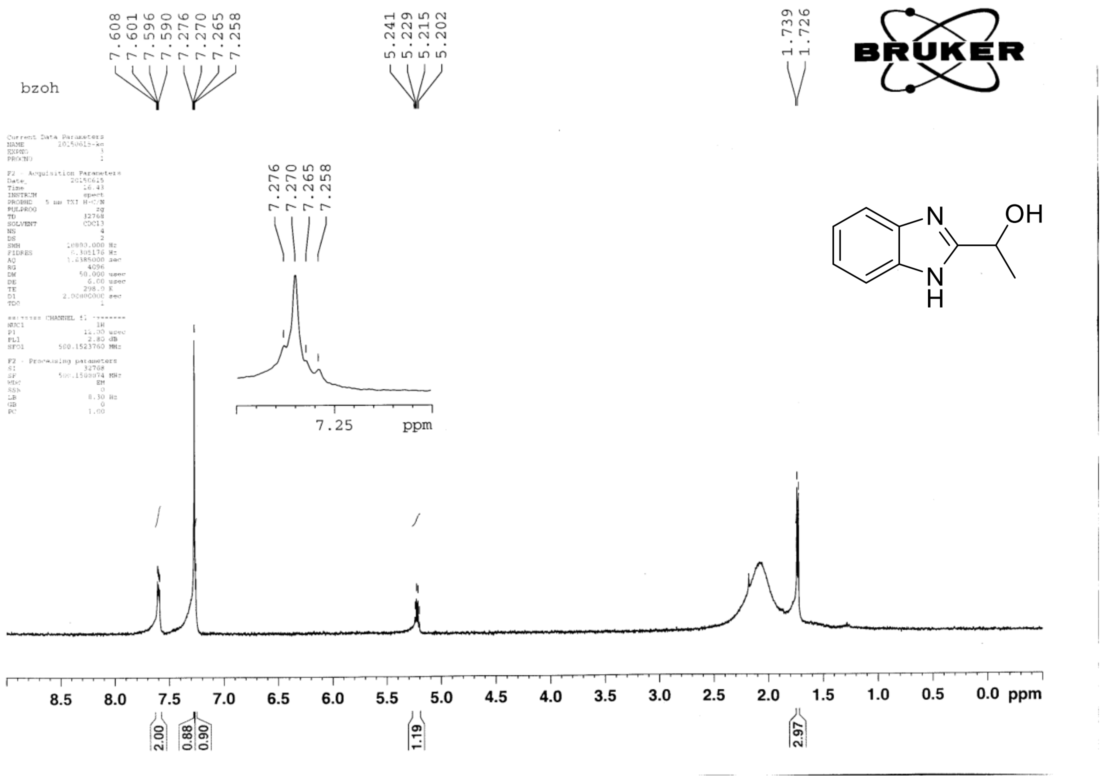

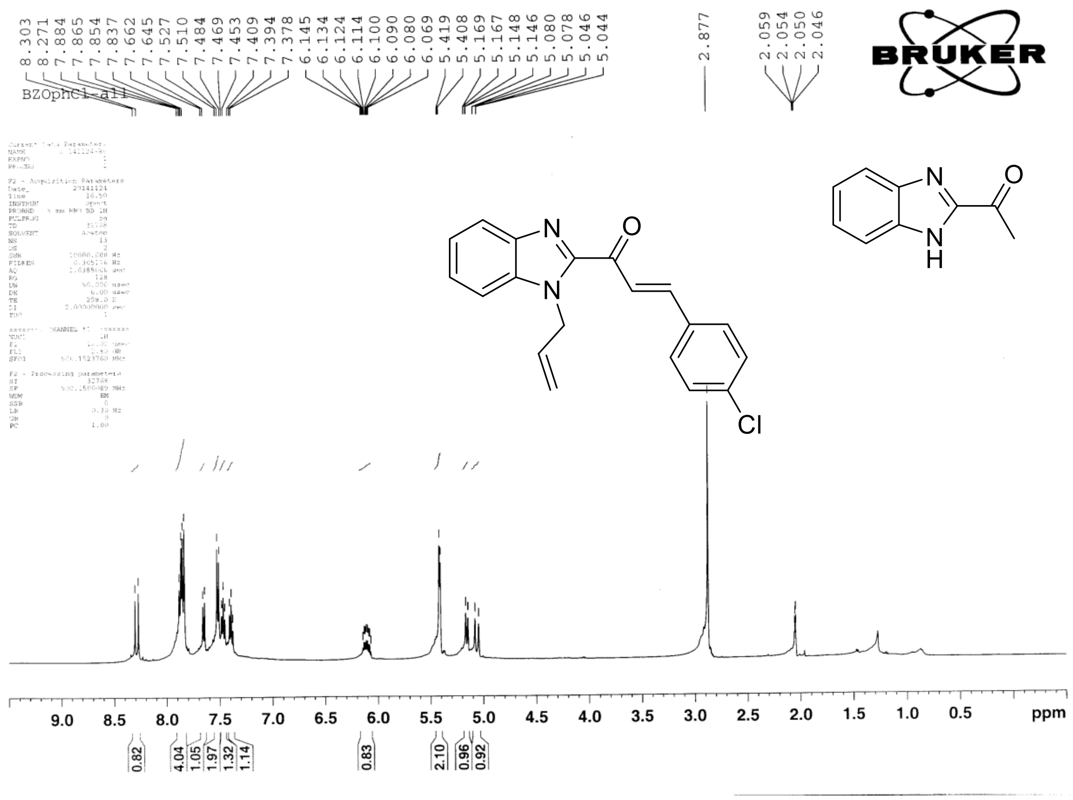

^{1}H-NMR spectrum of compound **19d**

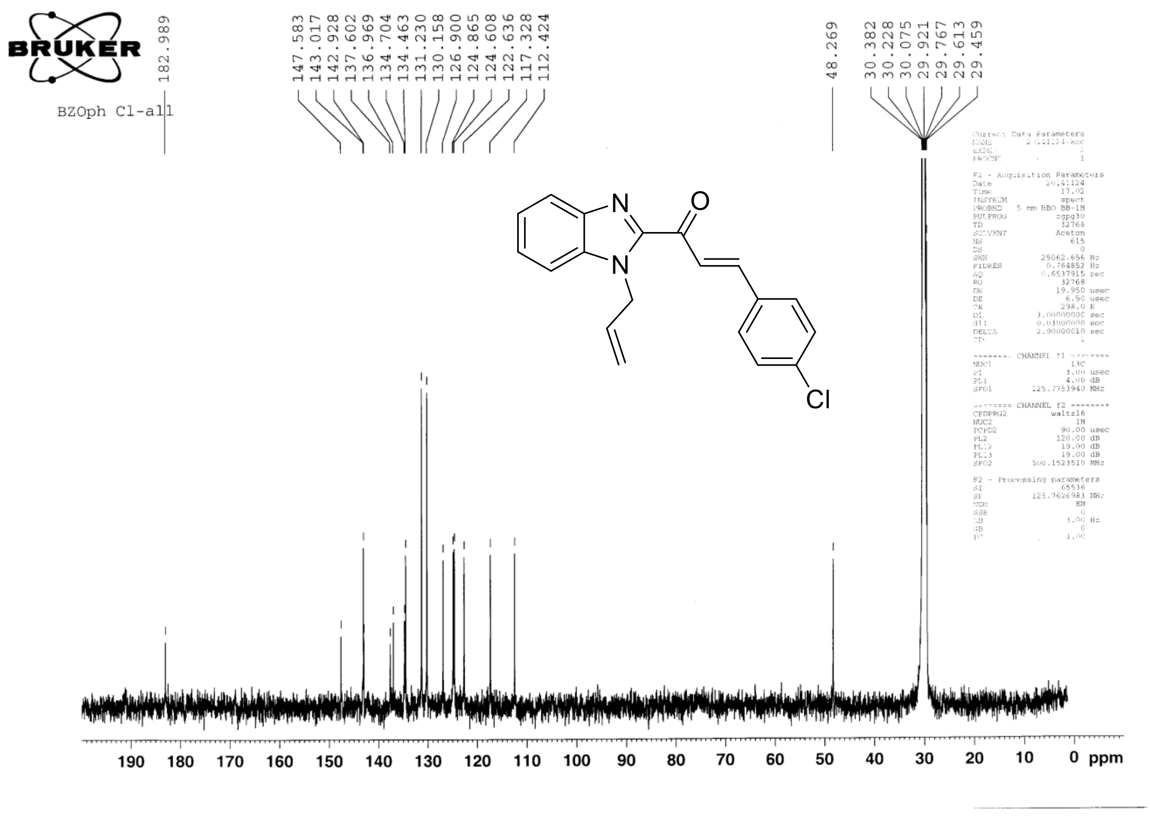

^{13}C-NMR spectrum of compound **19d**

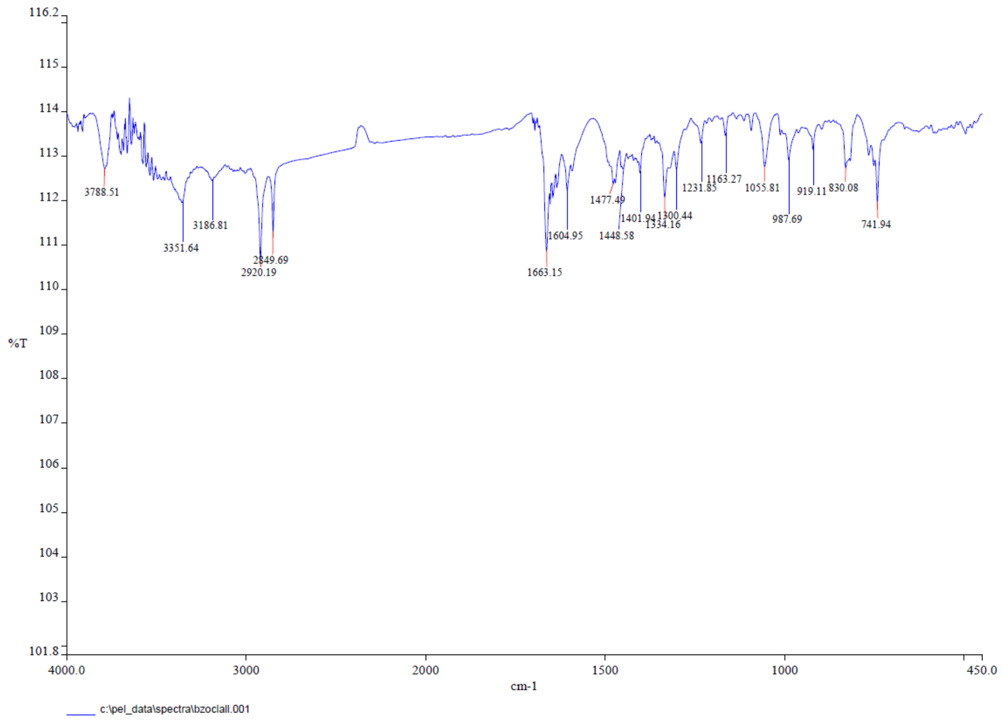

IR spectrum of compound **19d**

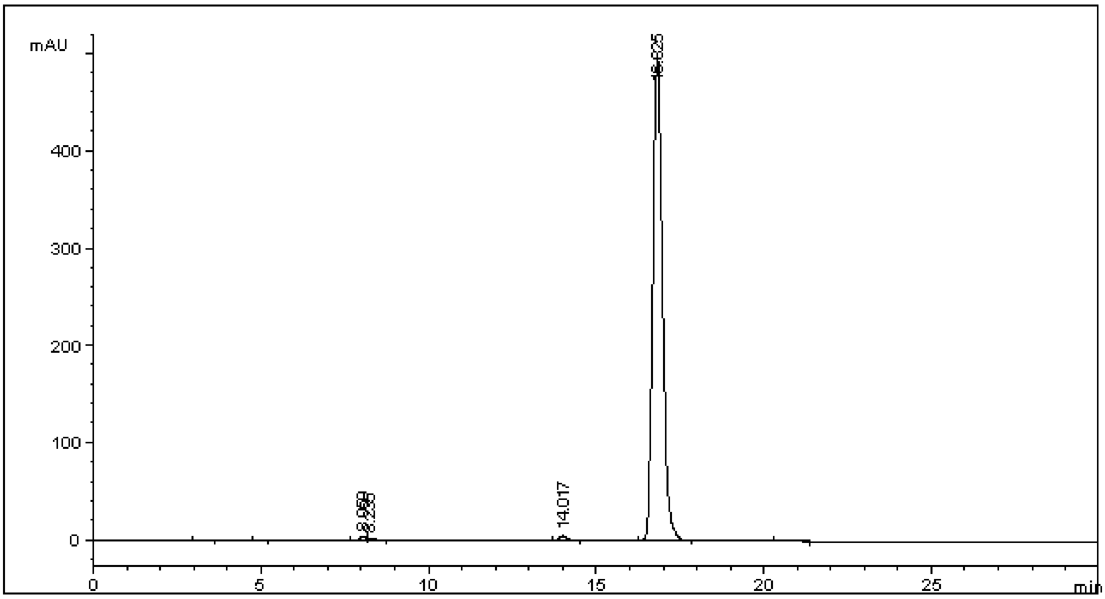

HPLC spectrum of compound **19d**

Peak #	RetTime [min]	Width [min]	Area mAU *s	Area %
1	8.05	0.2178	56.94883	0.5562
2	8.235	0.2043	36.42011	0.3557
3	14.017	0.2913	94.06382	0.9187
4	16.825	0.3131	10051.4	98.169

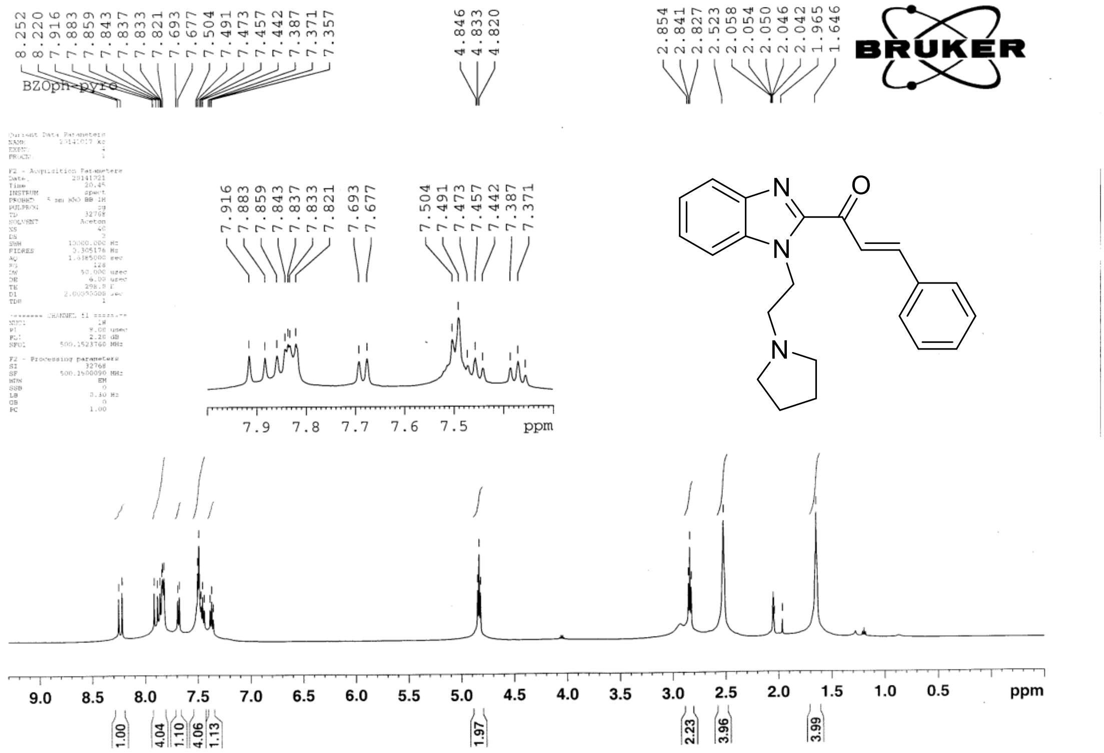

^{1}H-NMR spectrum of compound **20a**

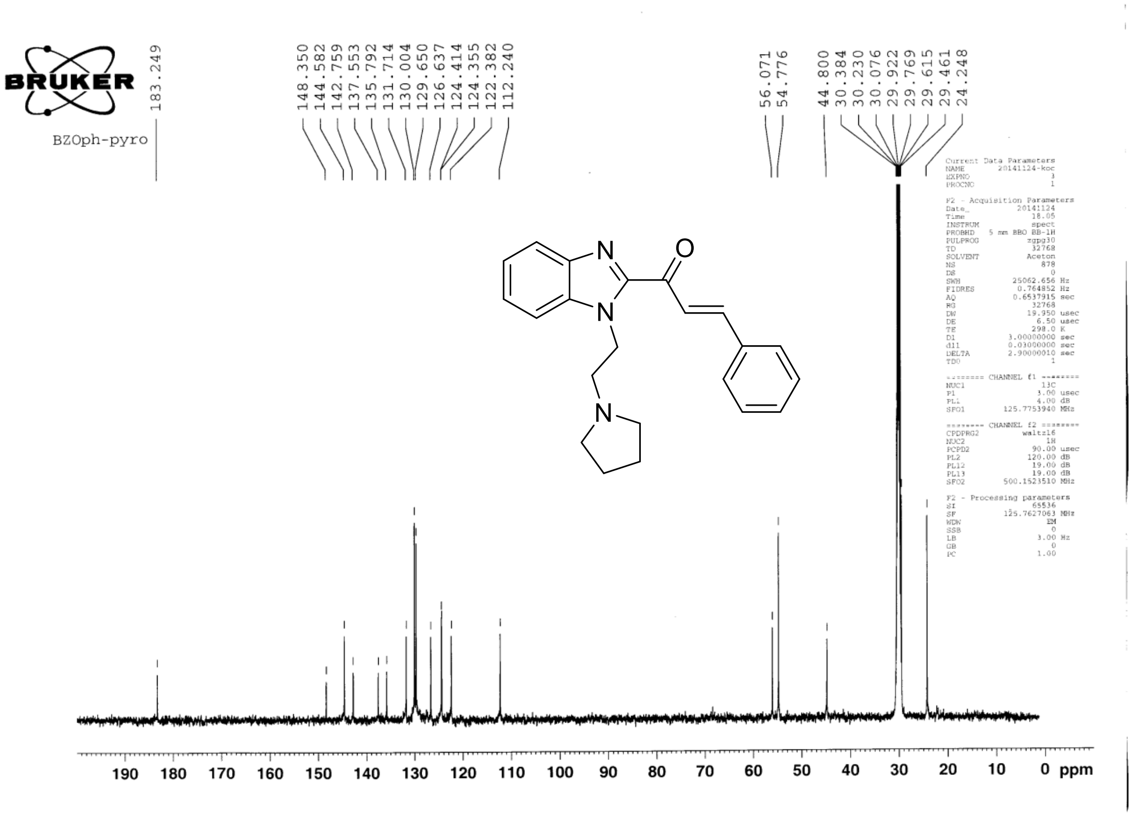

^{13}C-NMR spectrum of compound **20a**

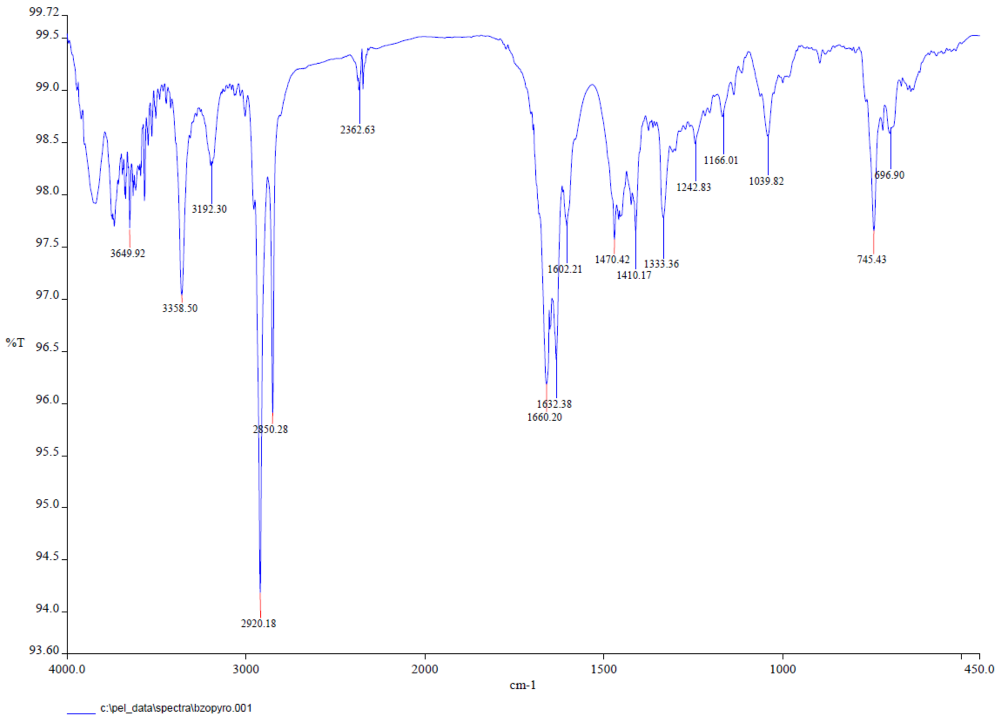

IR spectrum of compound **20a**

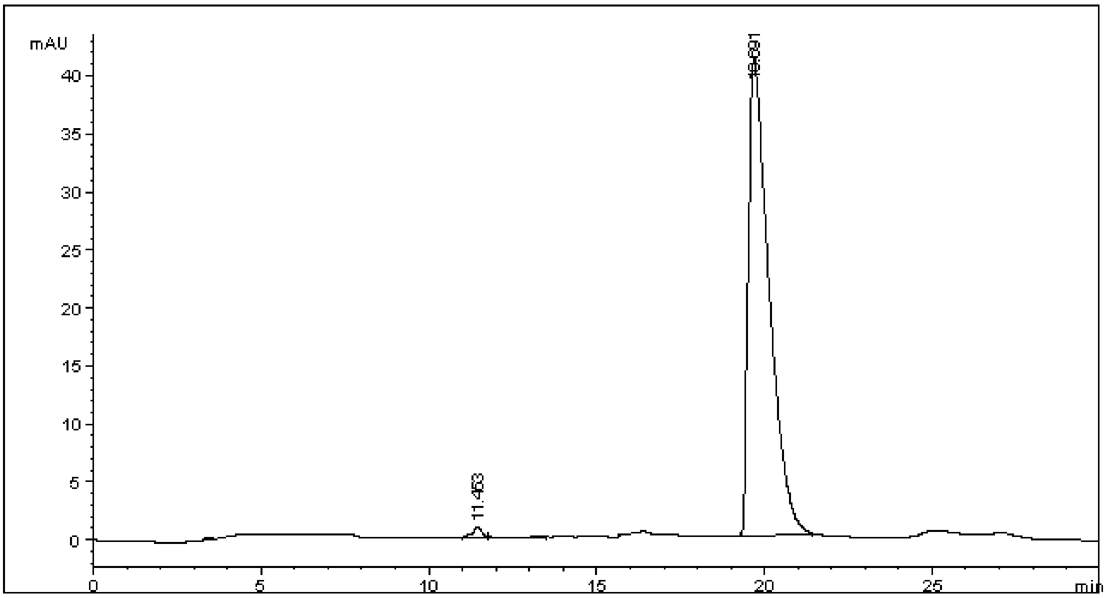

HPLC spectrum of compound **20a**

Peak #	RetTime [min]	Width [min]	Area mAU *s	Area %
1	11.453	0.3000	71.80160	3.8726
2	19.691	0.6477	1782.26953	96.1274

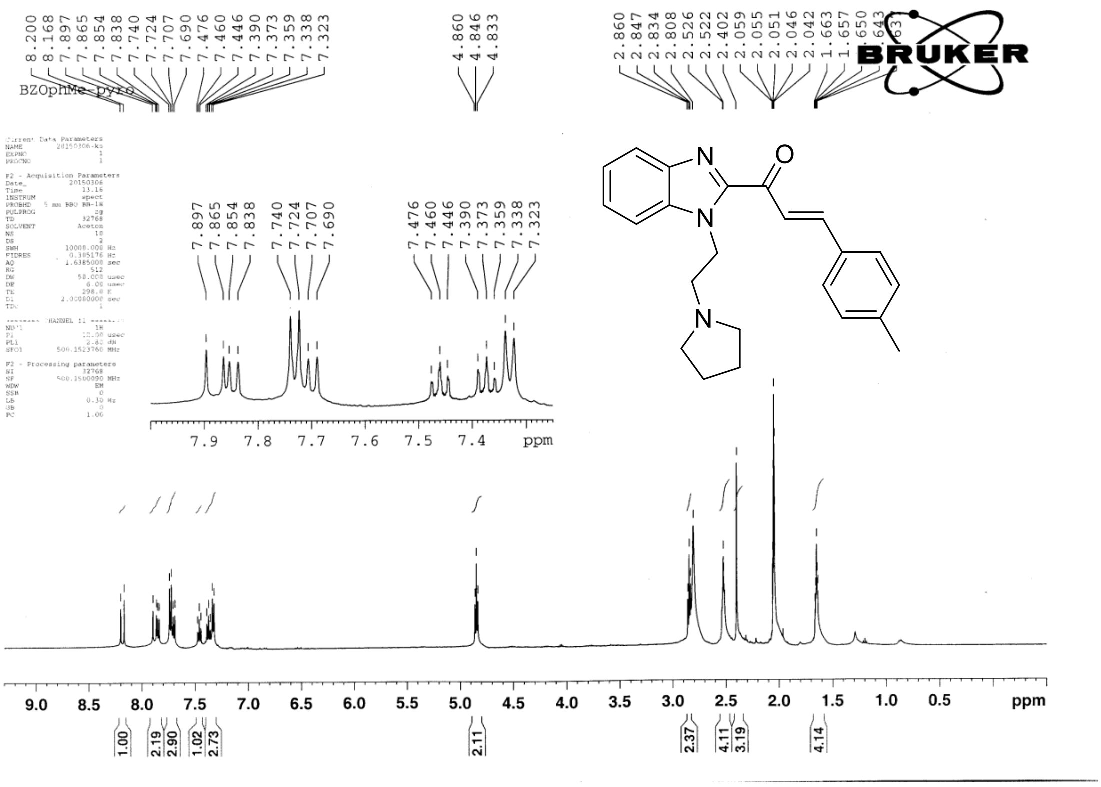

^{1}H-NMR spectrum of compound **20b**

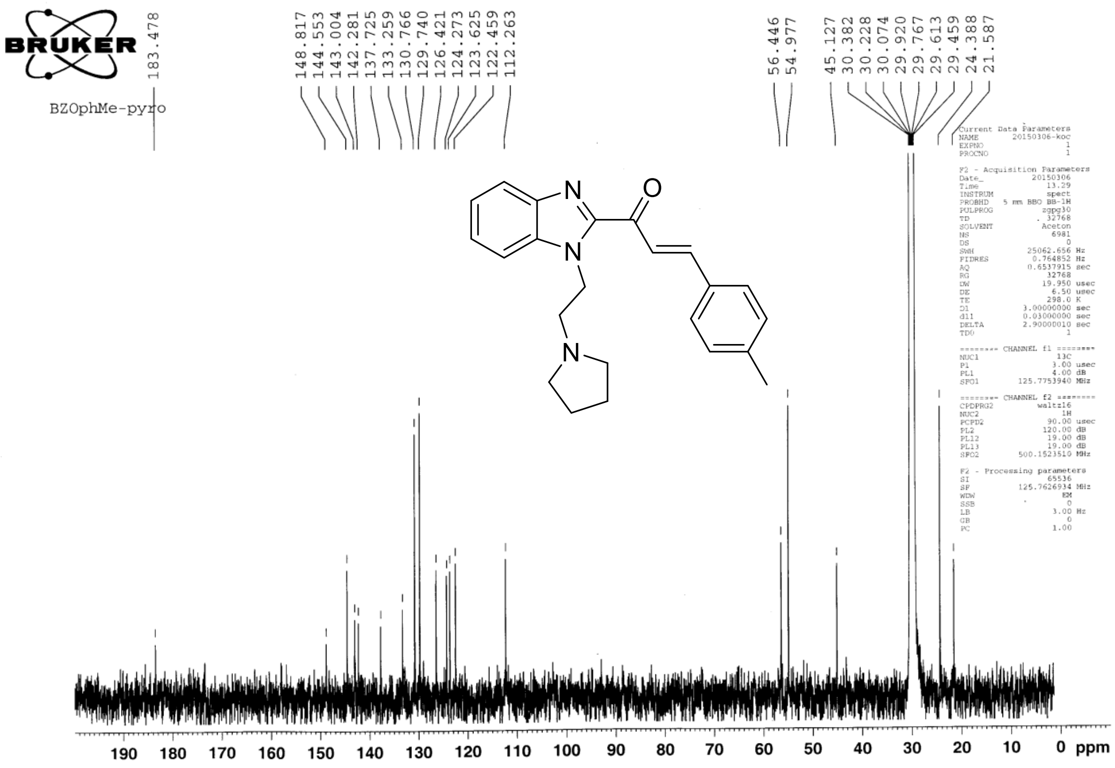

^{13}C-NMR spectrum of compound **20b**

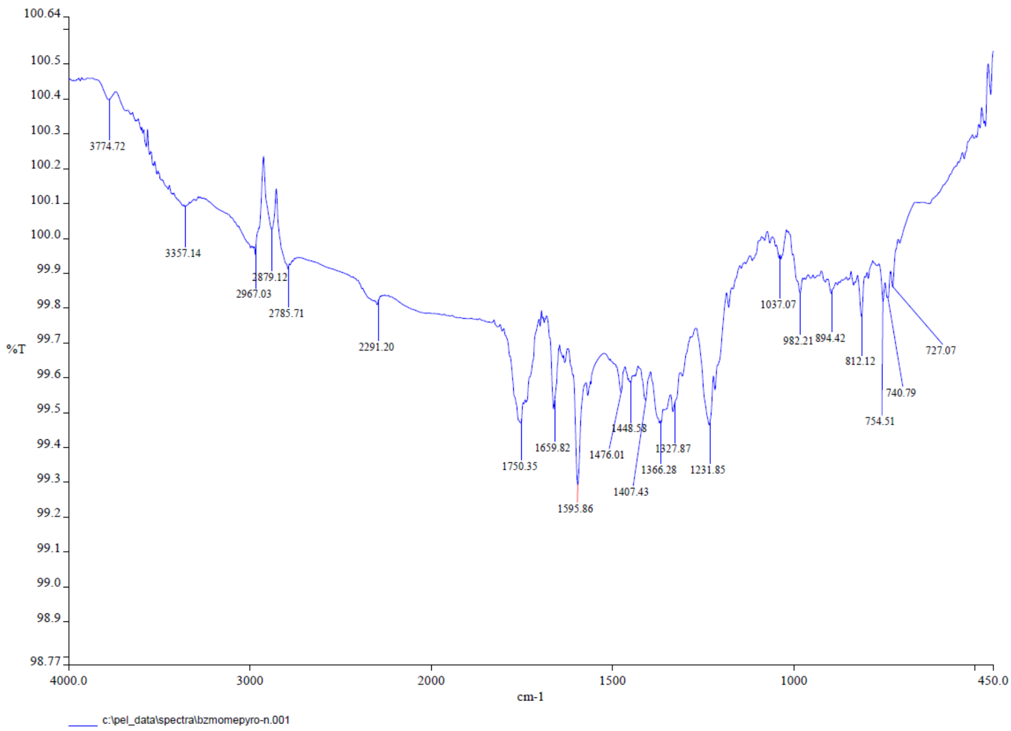

IR spectrum of compound **20b**

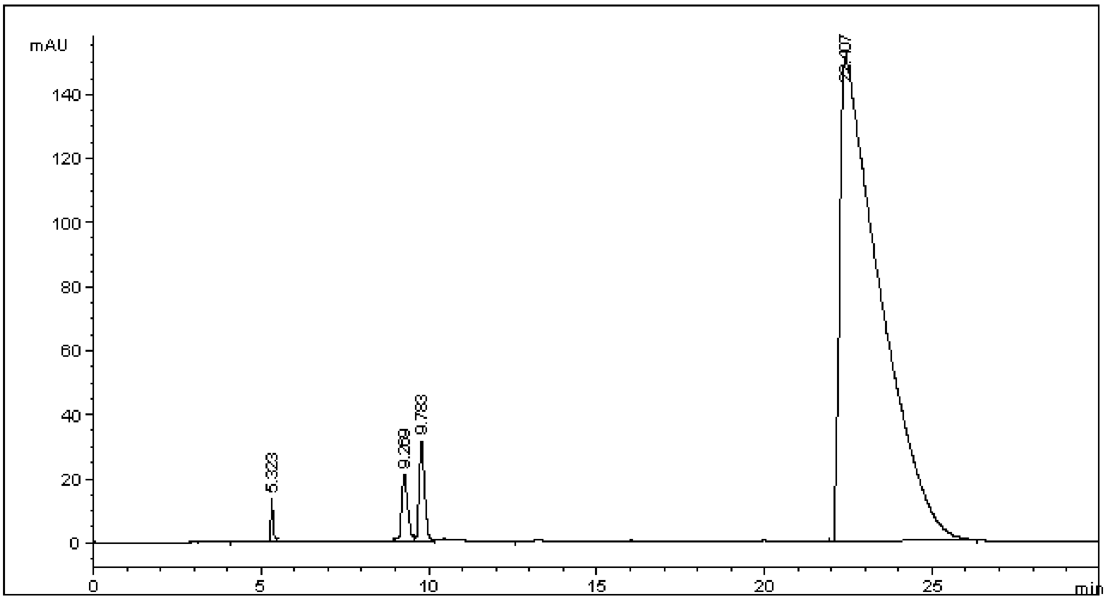

HPLC spectrum of compound **20b**

Peak #	RetTime [min]	Width [min]	Area mAU *s	Area %
1	5.323	0.0522	36.09950	0.2756
2	9.270	0.1684	180.59157	1.3785
3	9.783	0.1675	287.45895	2.1943
4	22.407	1.3899	12596.0	96.1516

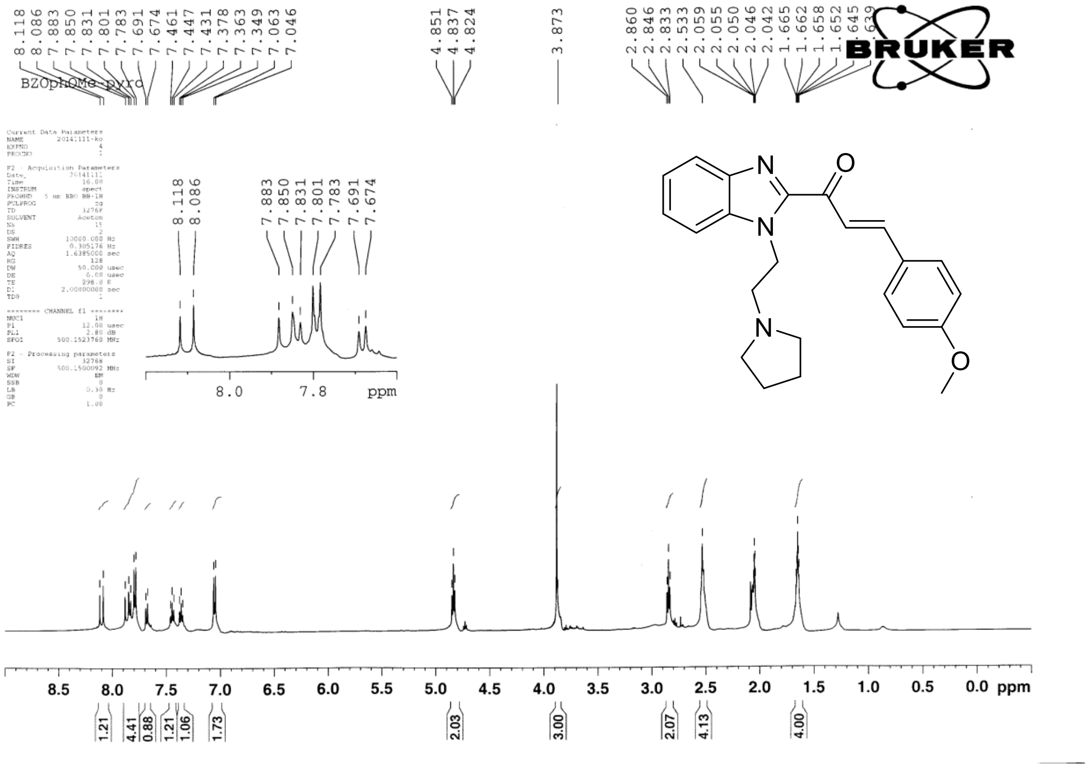

^{1}H-NMR spectrum of compound **20c**

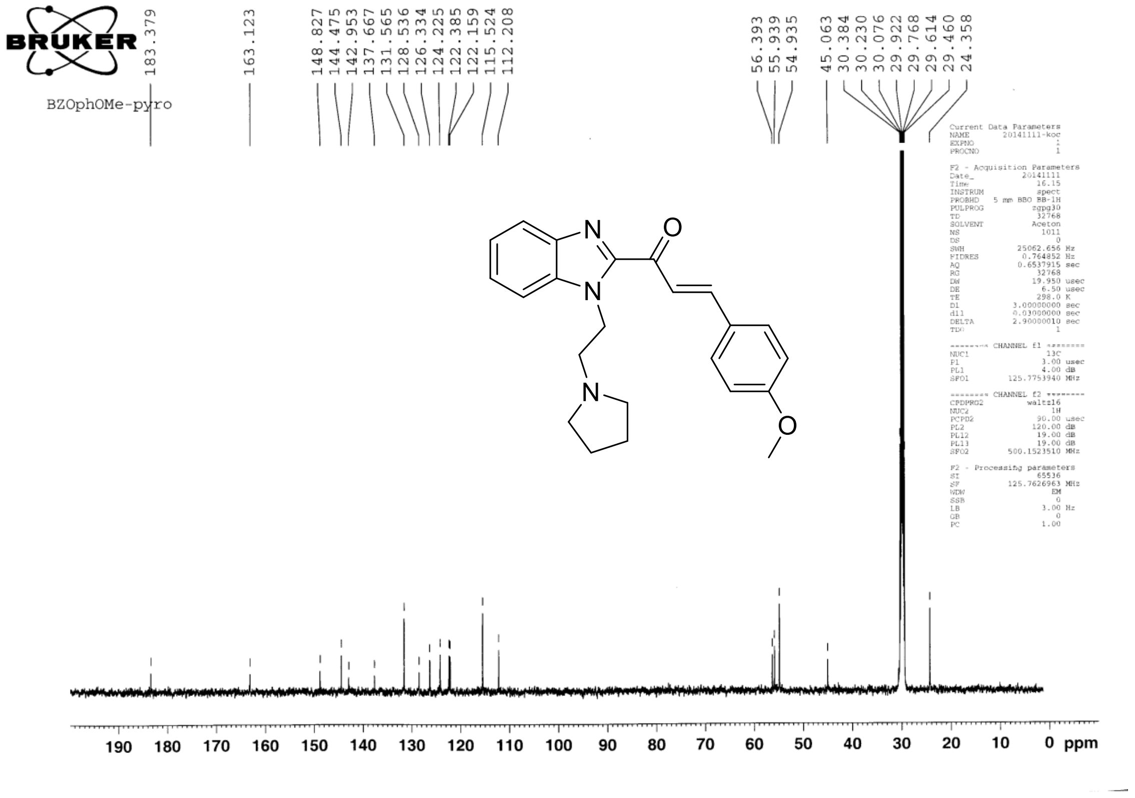

^{13}C-NMR spectrum of compound **20c**

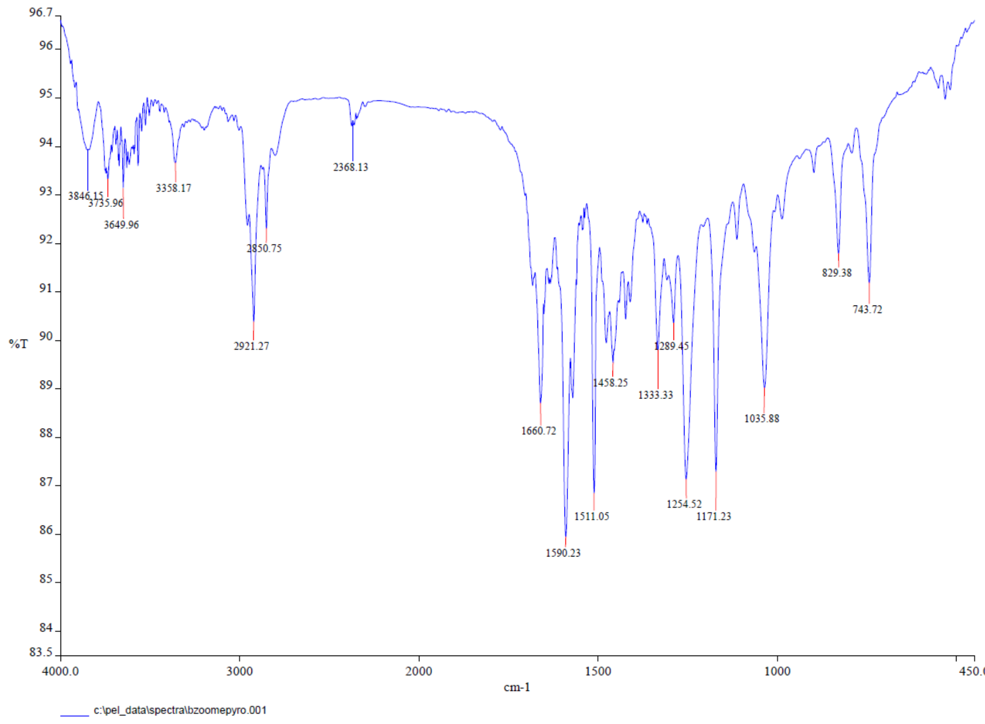

IR spectrum of compound **20c**

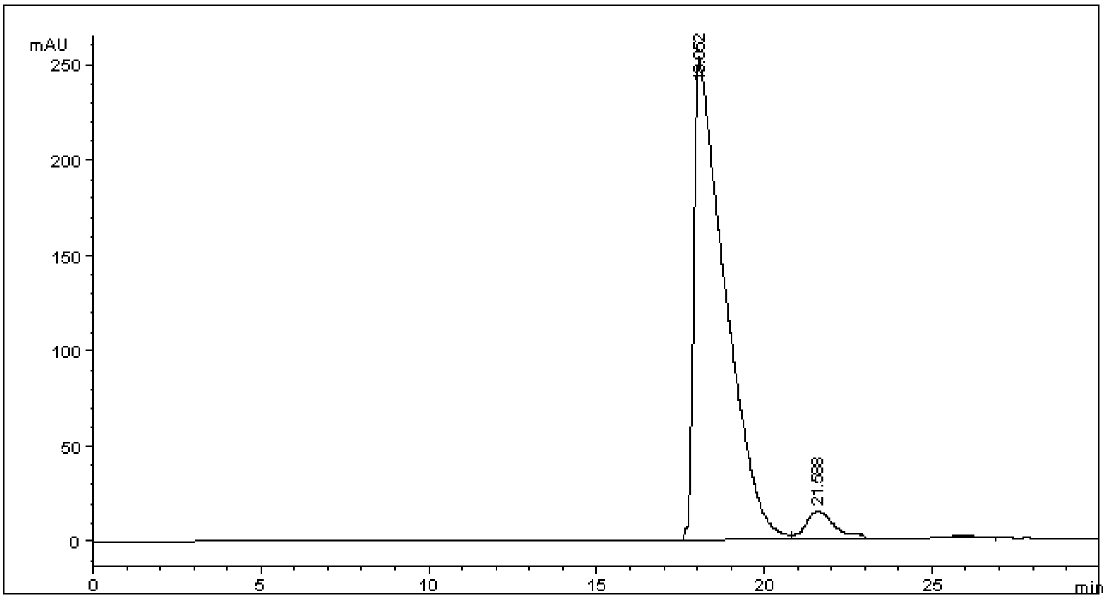

HPLC spectrum of compound **20c**

Peak<br>#	RetTime<br>[min]	Width<br>[min]	Area<br>mAU *s	Area<br>%
1	18.052	0.8788	16437.4	95.0057
2	21.588	0.9133	864.08667	4.9943

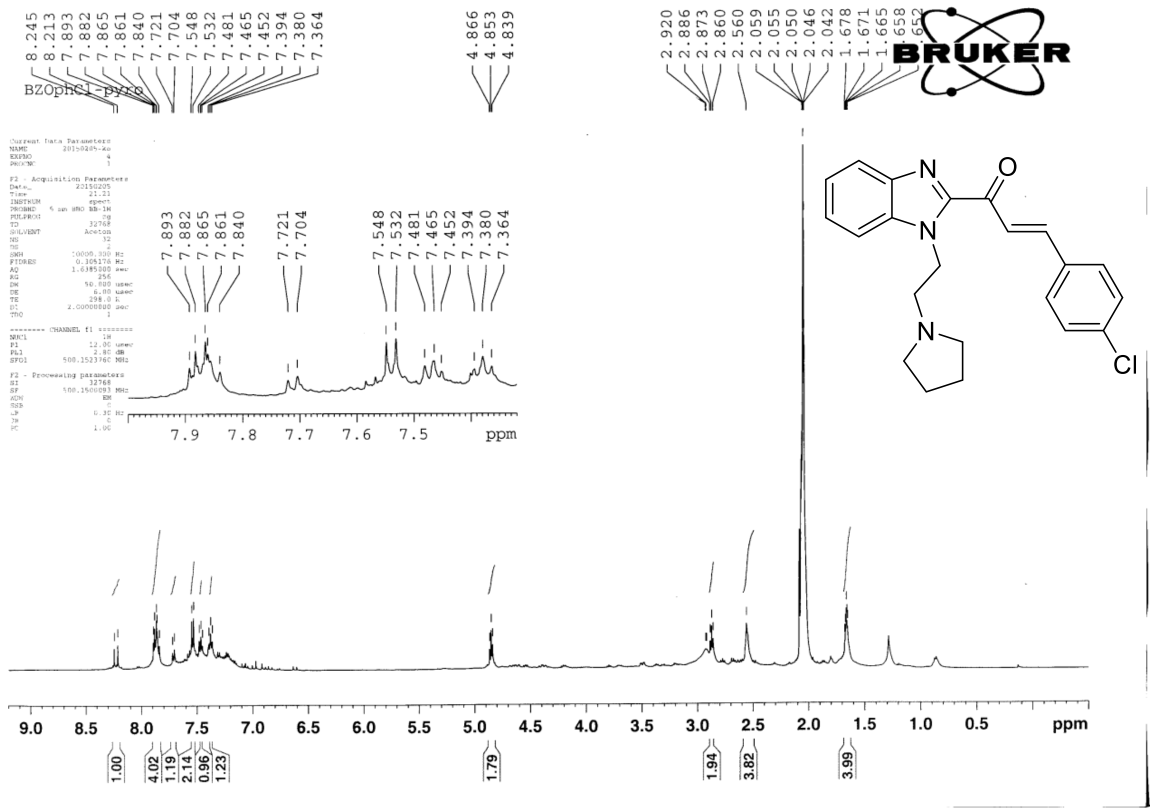

^{1}H-NMR spectrum of compound **20d**

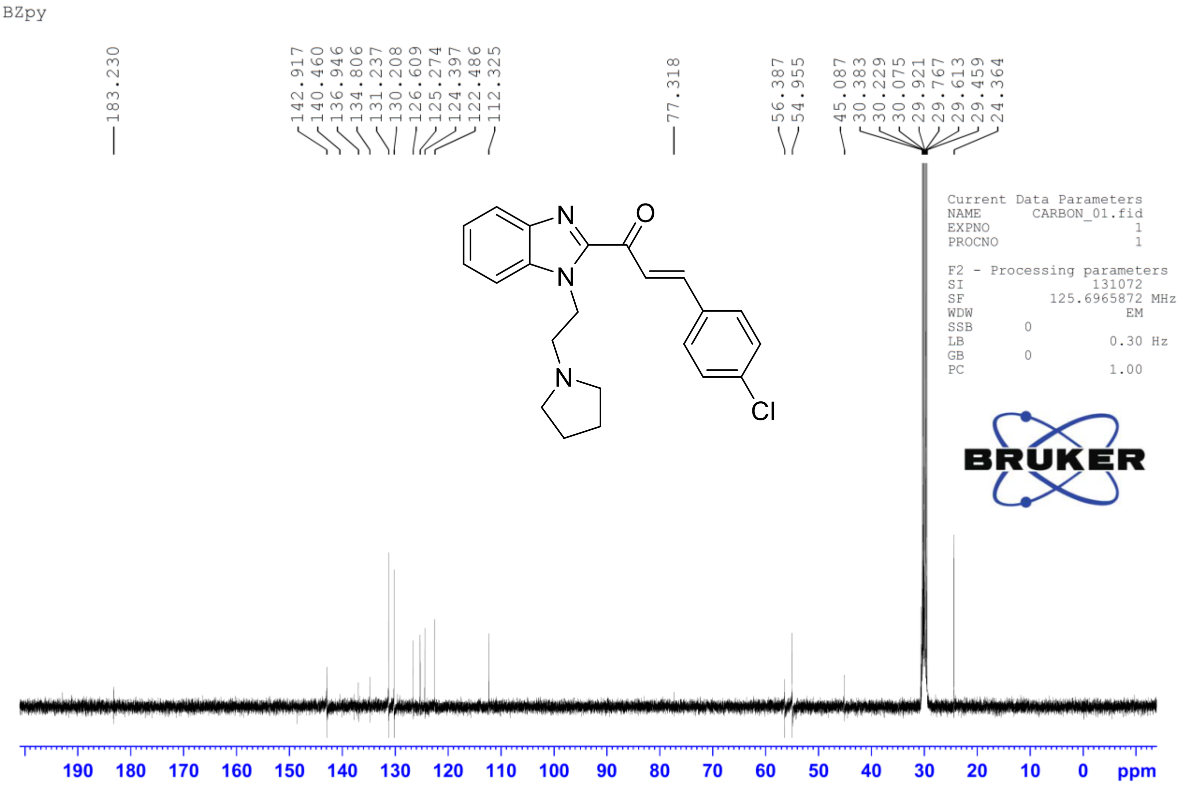

^{13}C-NMR spectrum of compound **20d**

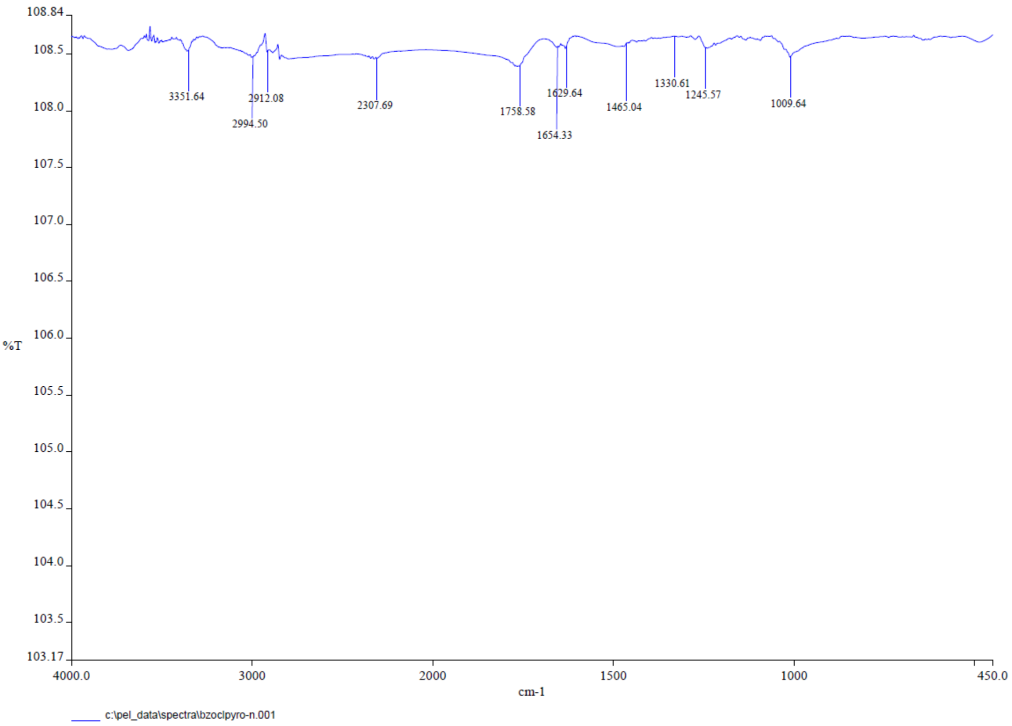

IR spectrum of compound **20d**

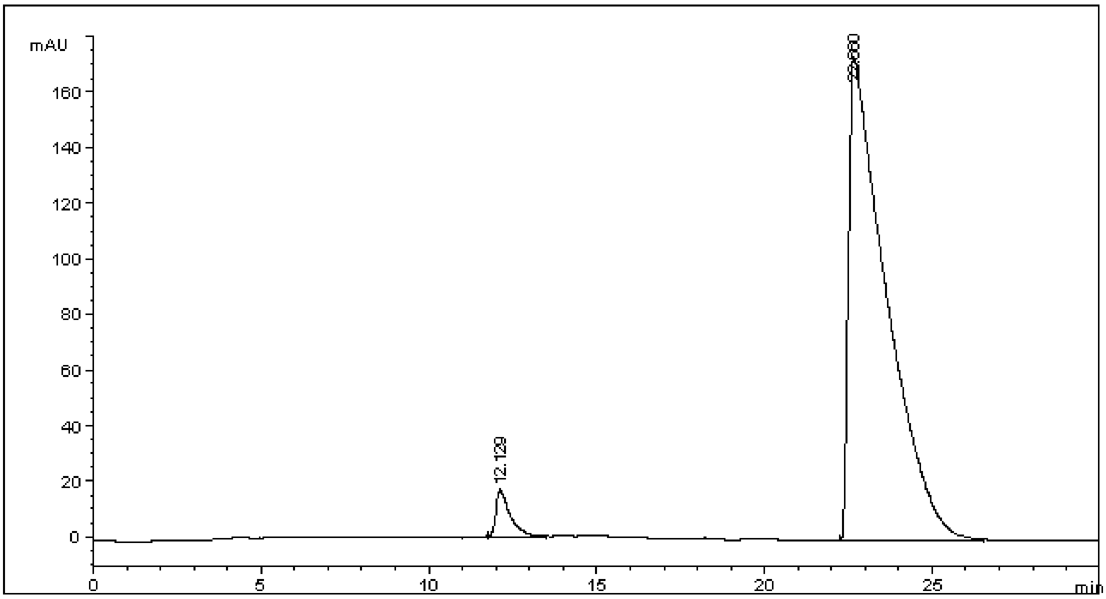

HPLC spectrum of compound **20d**

Peak #	RetTime [min]	Width [min]	Area mAU *s	Area %
1	12.129	0.4295	491.38254	3.5048
2	22.660	1.0394	13528.9	96.4953

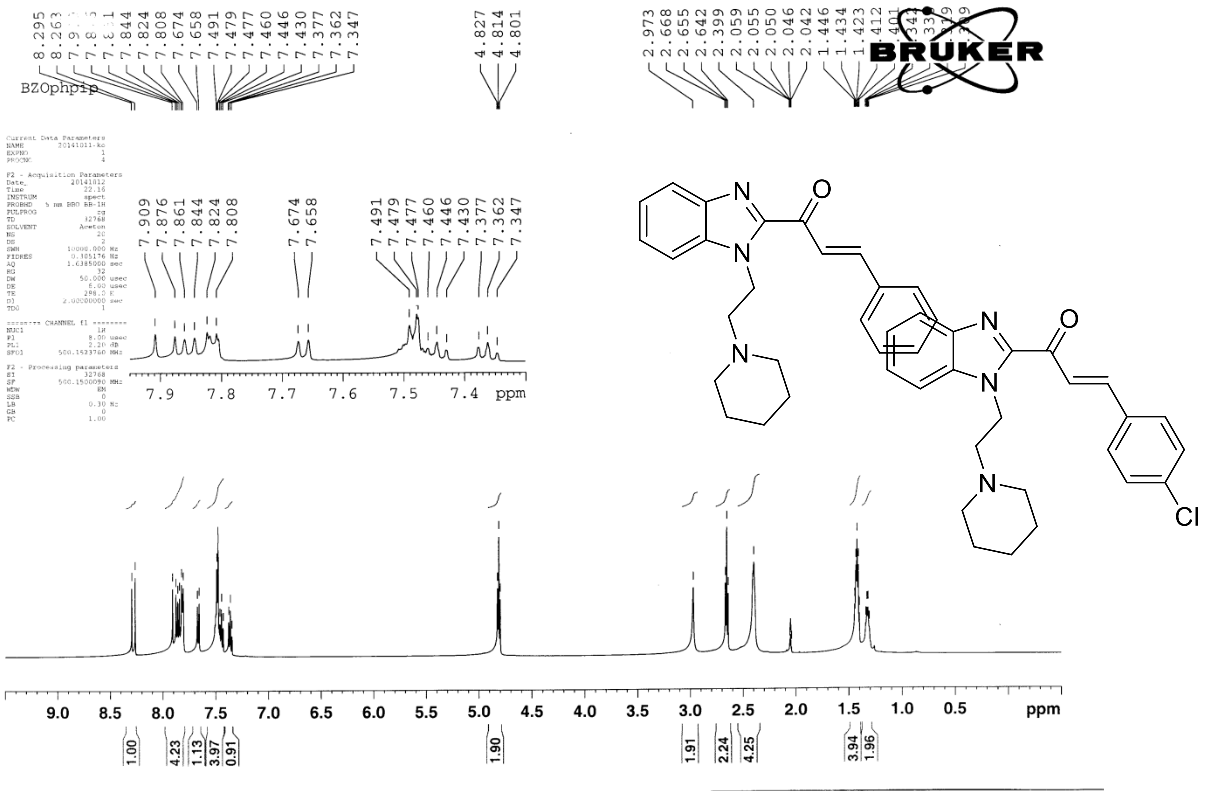

^{1}H-NMR spectrum of compound **21a**

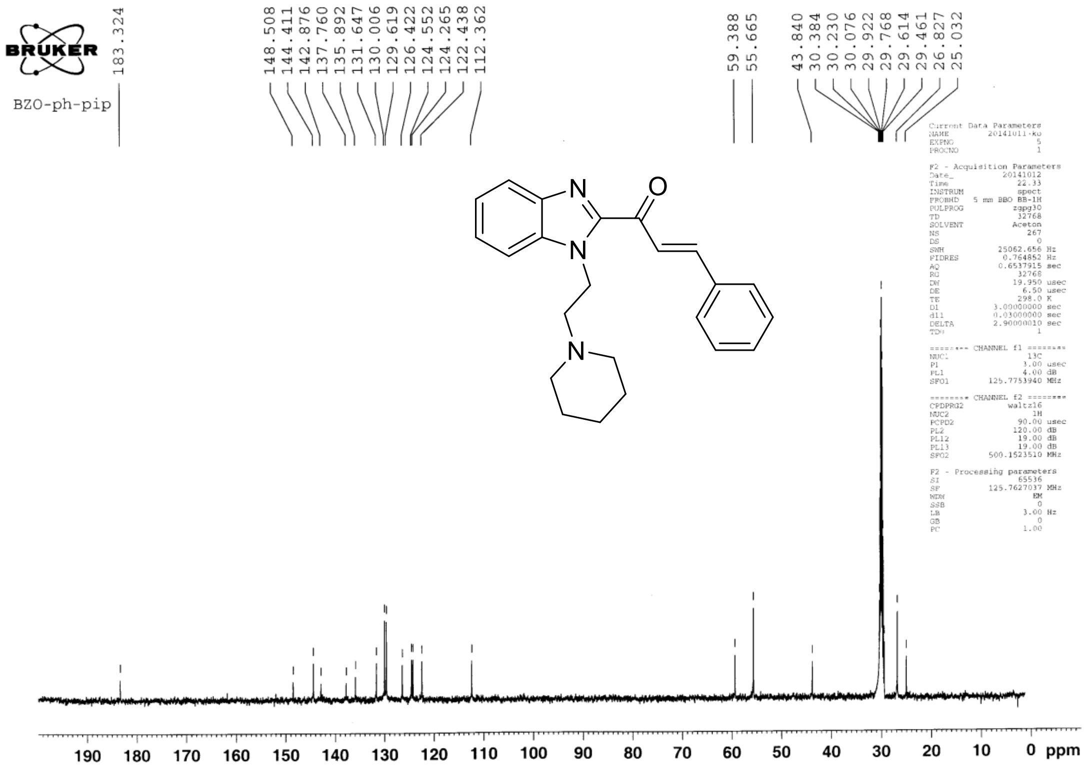

^{13}C-NMR spectrum of compound **21a**

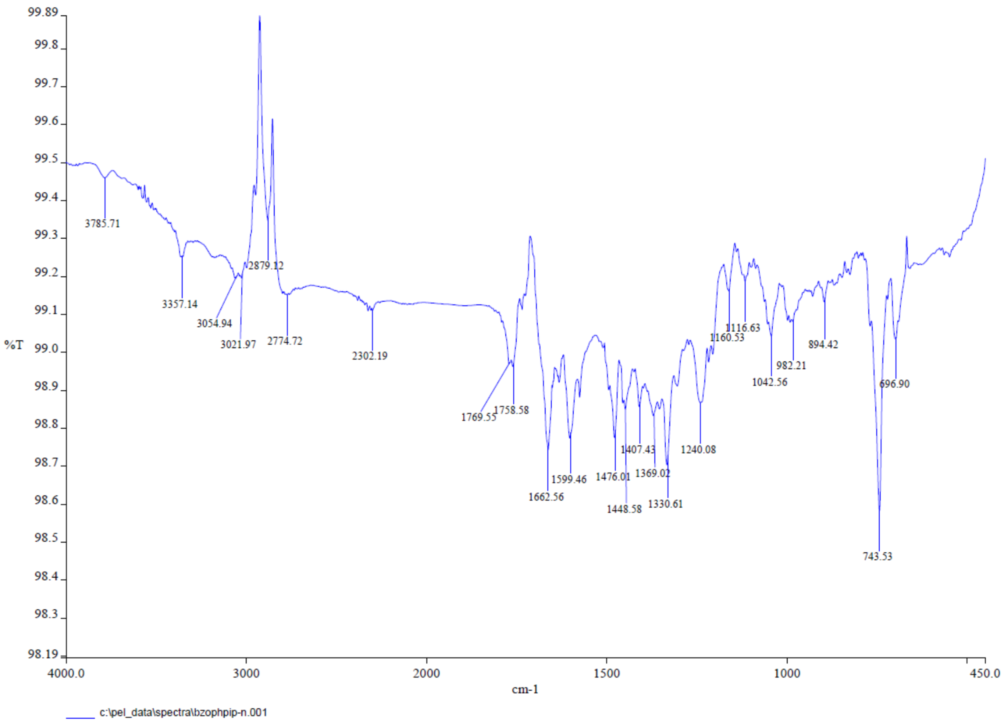

IR spectrum of compound **21a**

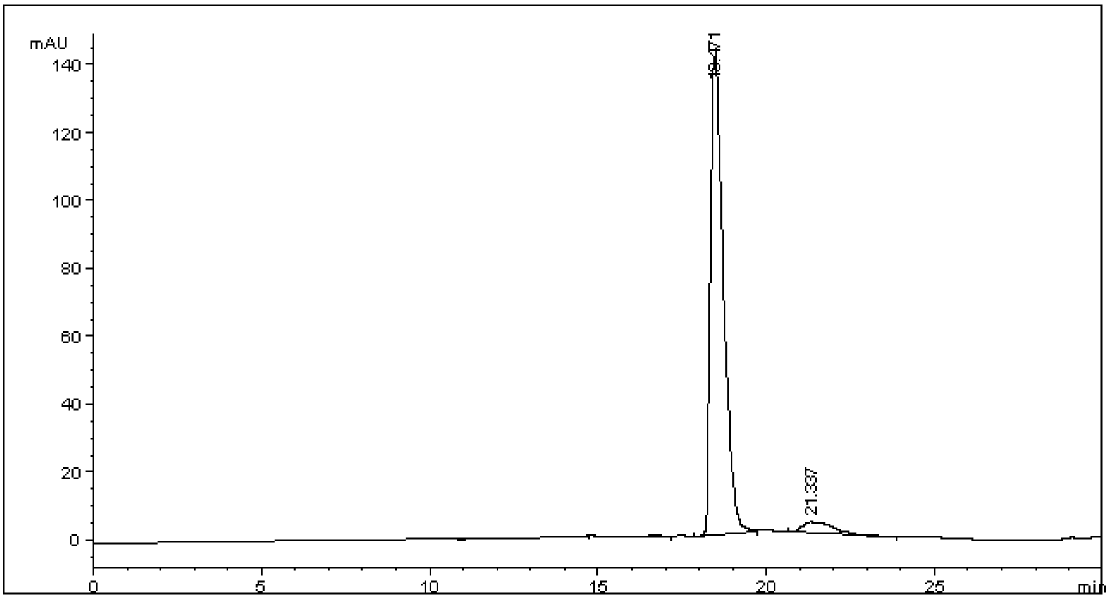

HPLC spectrum of compound **21a**

Peak<br>\#	RetTime<br>[min]	Width<br>[min]	Area<br>mAU *s	Area<br>%
1	18.471	0.4193	3837.38745	95.0169
2	21.337	0.9008	201.24811	4.9831

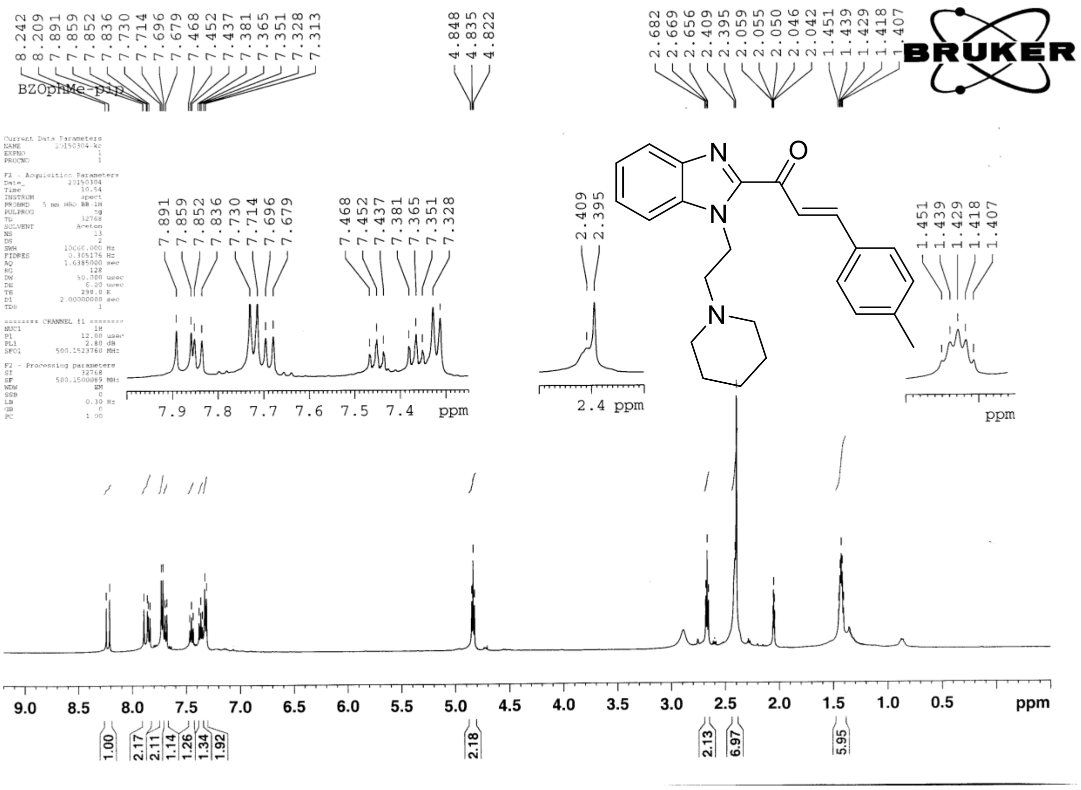

^{1}H-NMR spectrum of compound **21b**

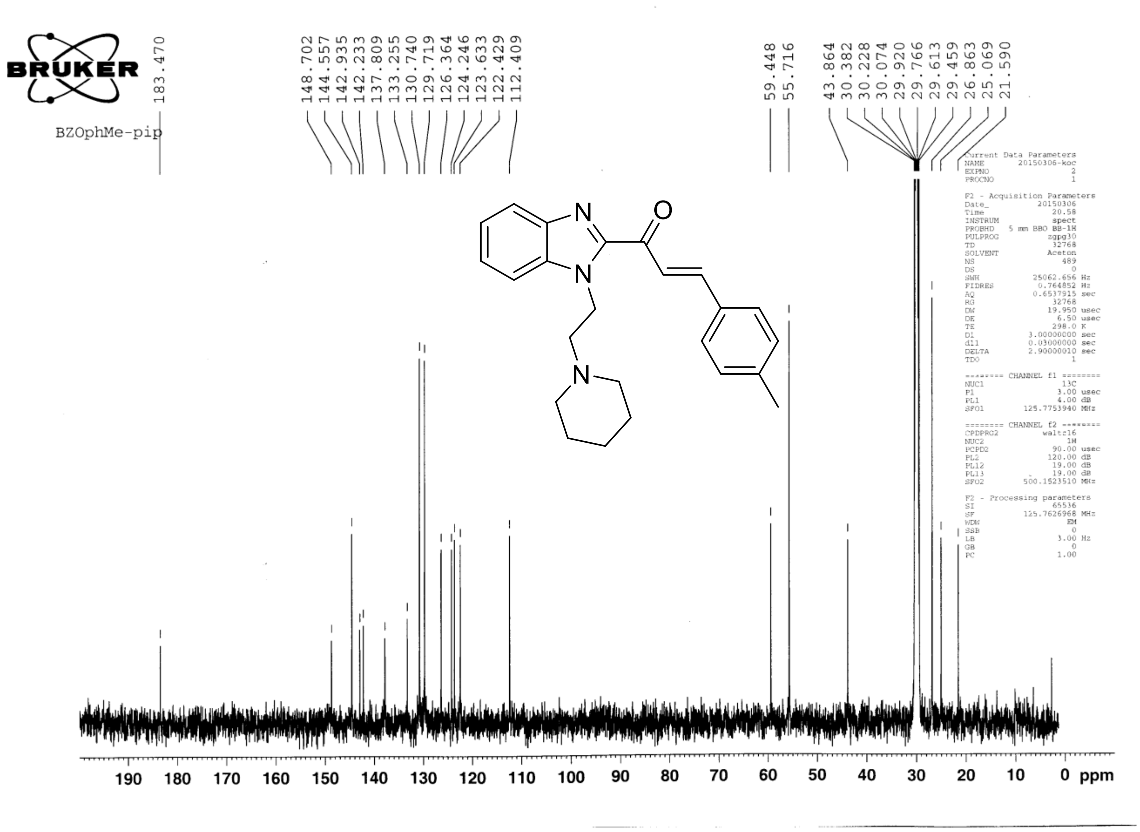

^{13}C-NMR spectrum of compound **21b**

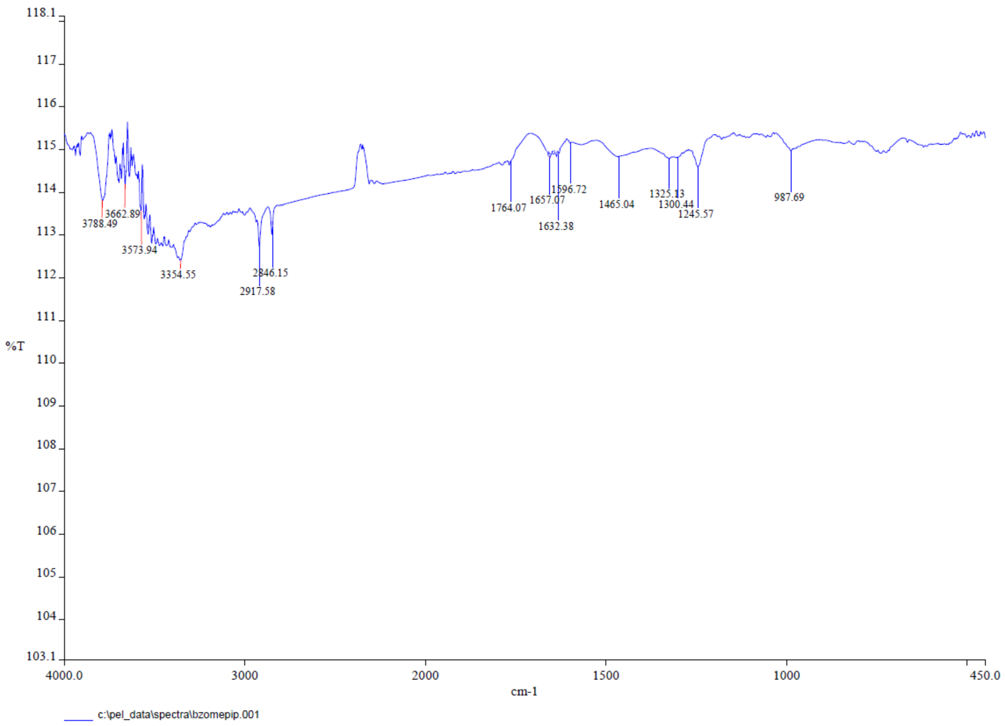

IR spectrum of compound **21b**

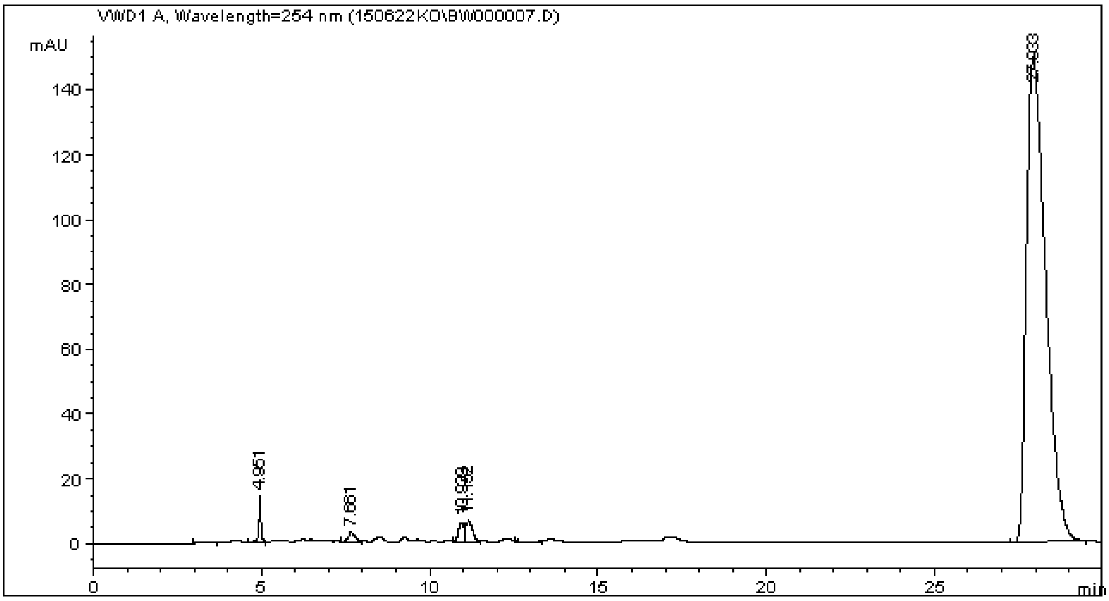

HPLC spectrum of compound **21b**

Peak #	RetTime [min]	Width [min]	Area mAU *s	Area %
1	4.951	0.0699	68.29758	1.0947
2	7.661	0.1966	38.23409	0.6129
3	10.939	0.1666	64.38687	1.0320
4	11.152	0.2067	91.49991	1.4666
5	27.933	0.6205	5976.29102	95.7937

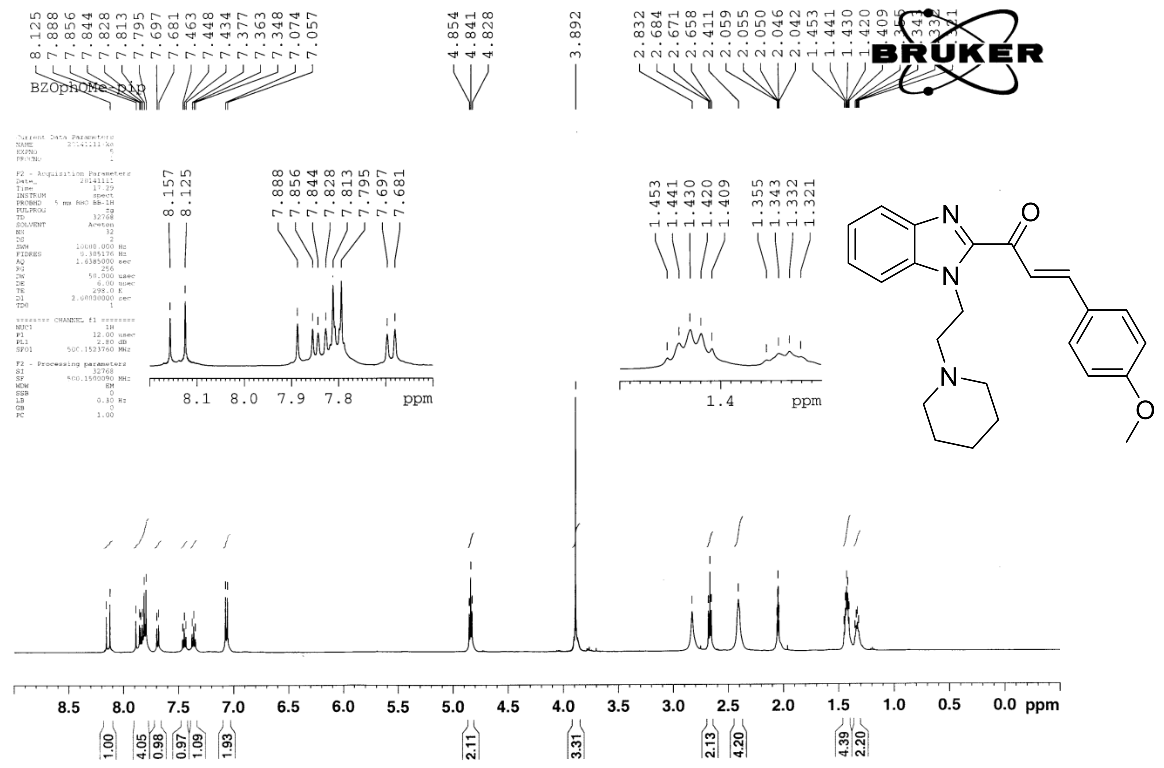

^{1}H-NMR spectrum of compound **21c**

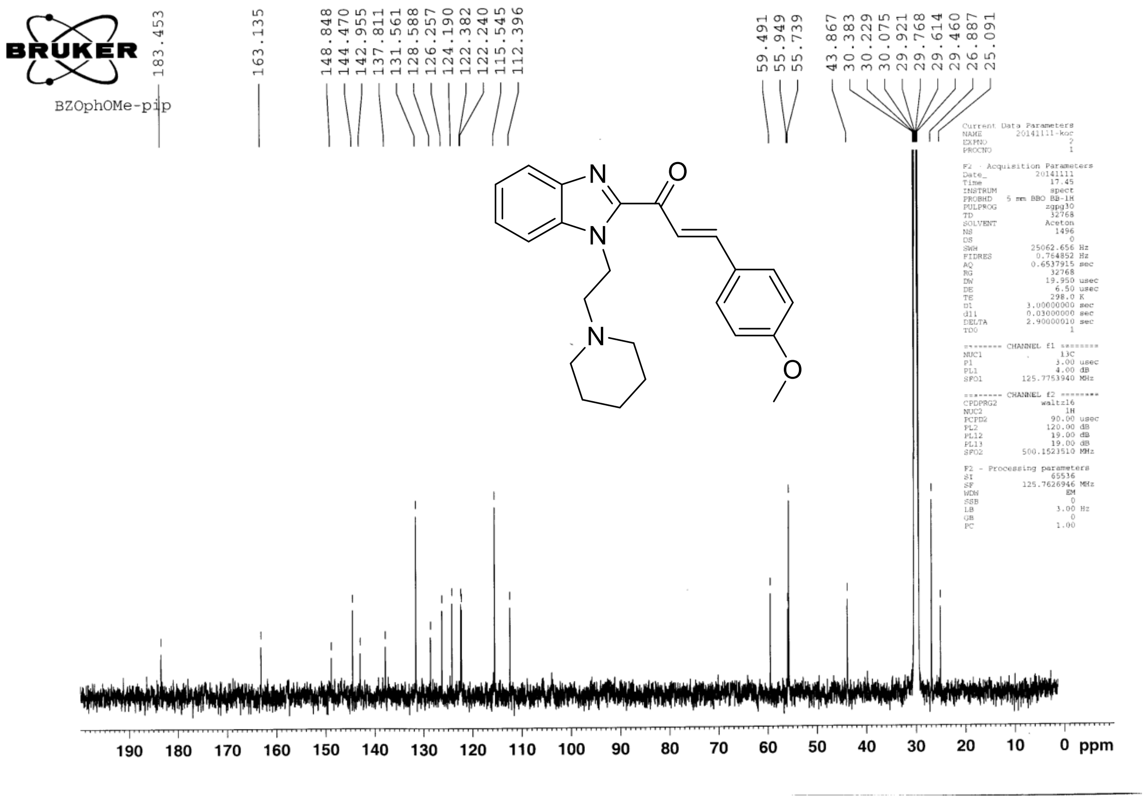

^{13}C-NMR spectrum of compound **21c**

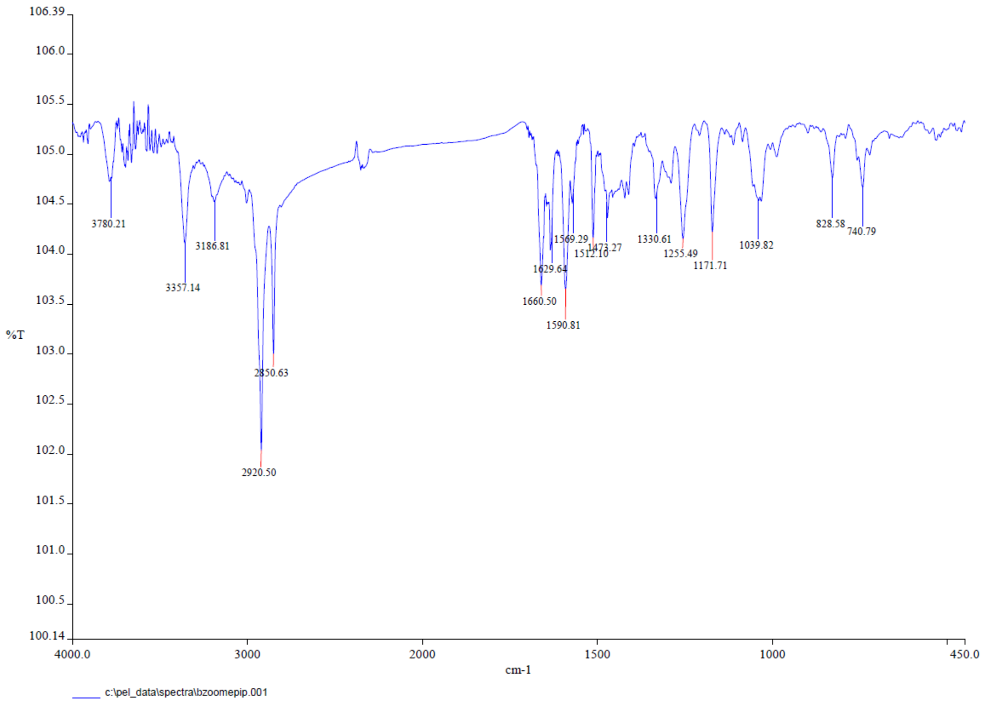

IR spectrum of compound **21c**

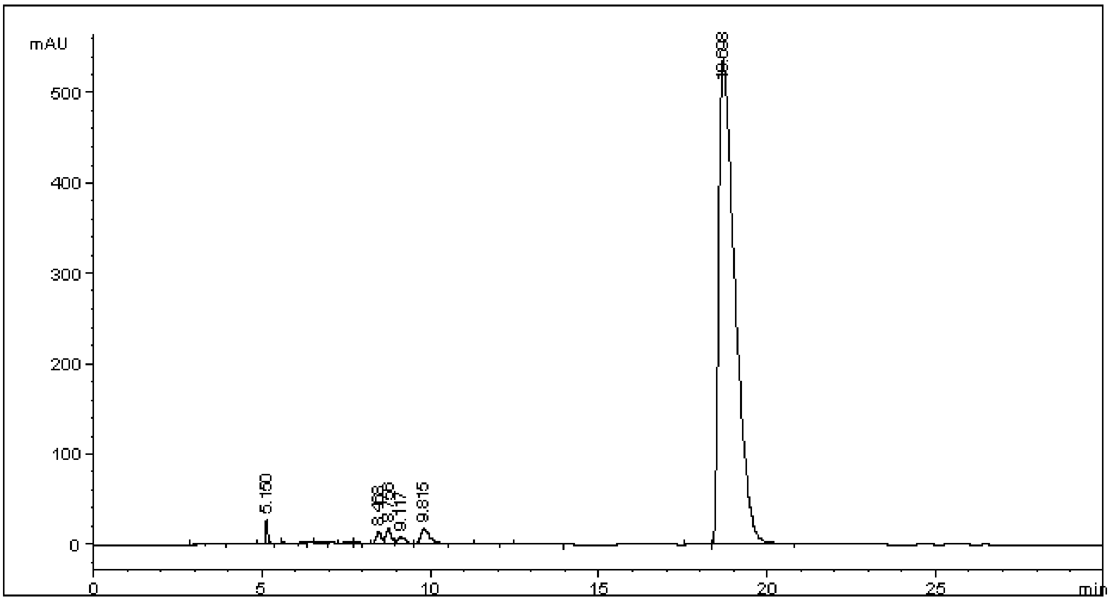

HPLC spectrum of compound **21c**

Peak #	RetTime [min]	Width [min]	Area mAU *s	Area %
1	5.150	0.0654	120.31086	0.6396
2	8.468	0.1586	143.72900	0.7641
3	8.756	0.1657	182.23738	0.9688
4	9.117	0.2214	113.33360	0.6025
5	9.815	0.2669	299.16333	1.5904
6	18.698	0.4951	17951.6	95.4346

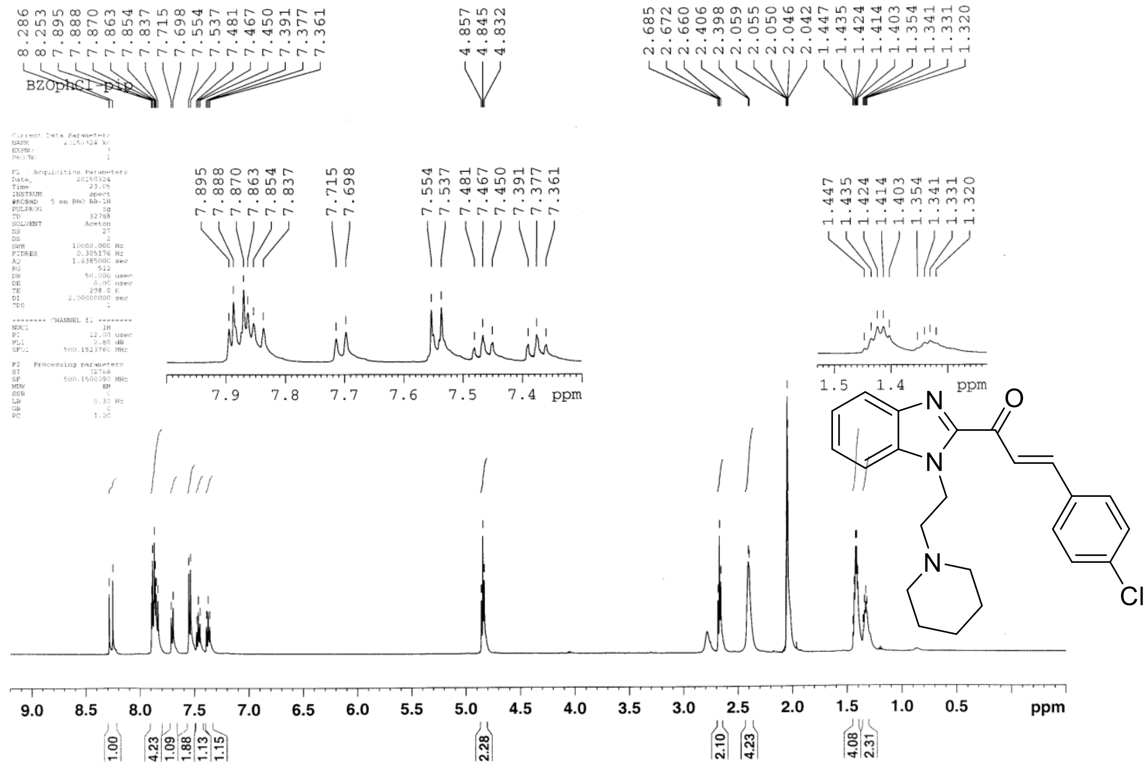

^{1}H-NMR spectrum of compound **21d**

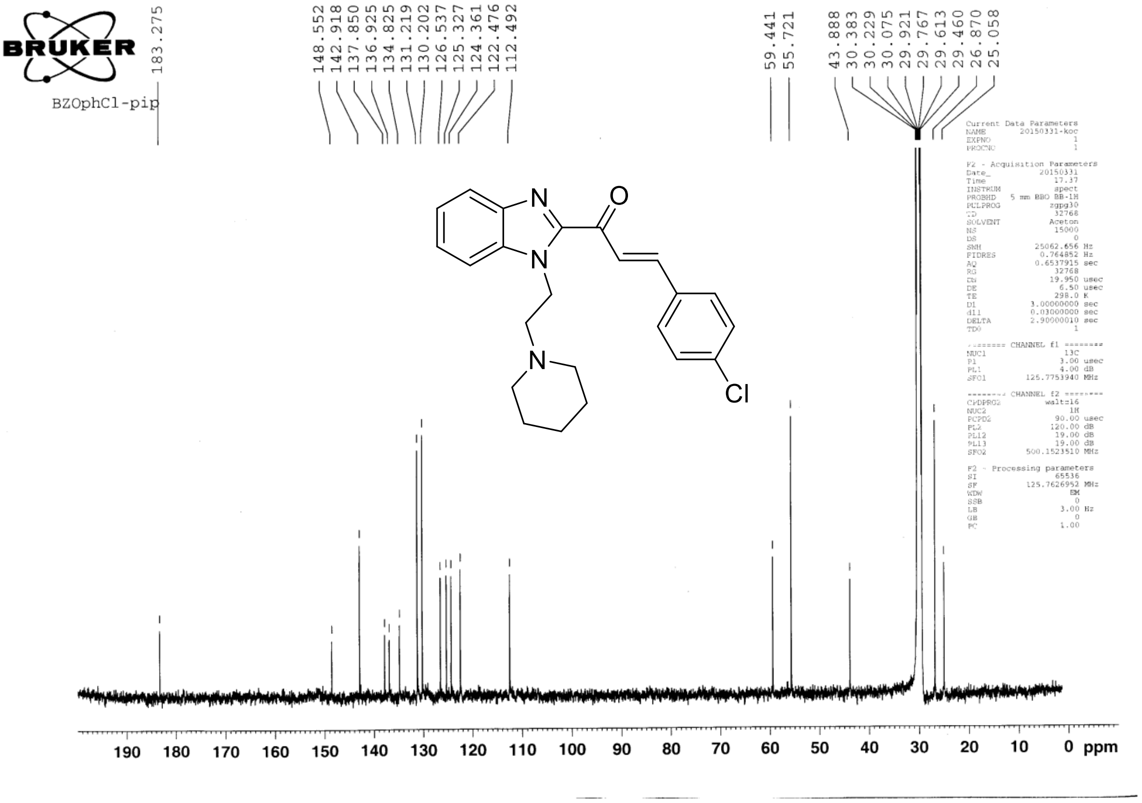

^{13}C-NMR spectrum of compound **21d**

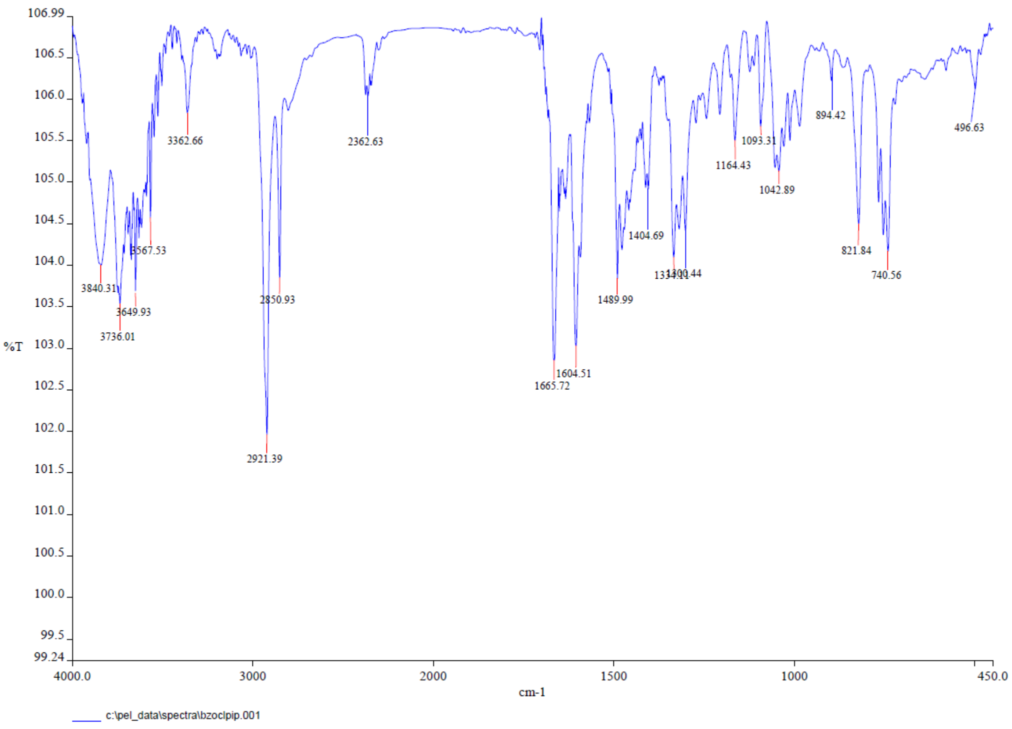

IR spectrum of compound **21d**

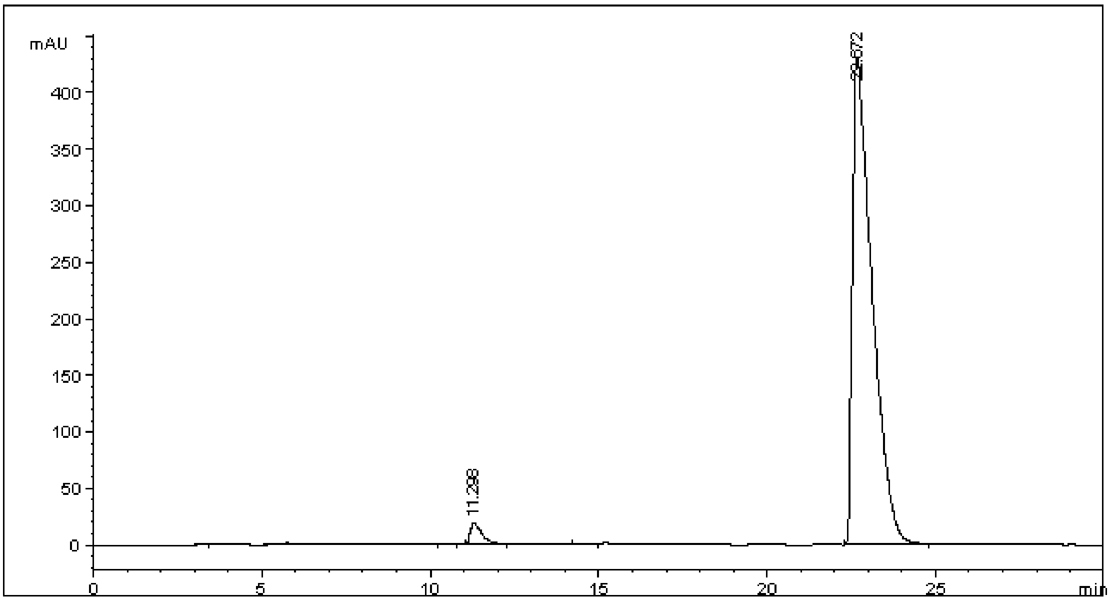

HPLC spectrum of compound **21d**

Peak #	RetTime [min]	Width [min]	Area mAU *s	Area %
1	11.298	0.3362	439.36765	2.4527
2	22.672	0.5824	17474.1	97.5473

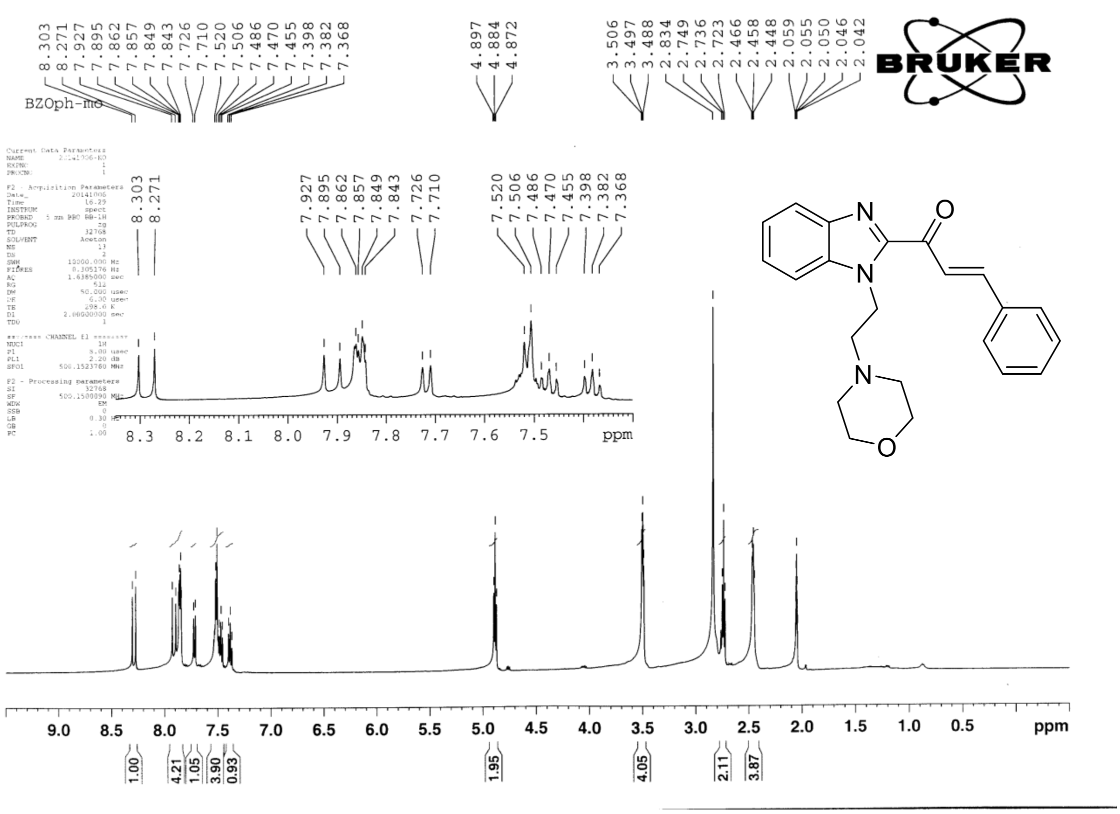

^{1}H-NMR spectrum of compound **22a**

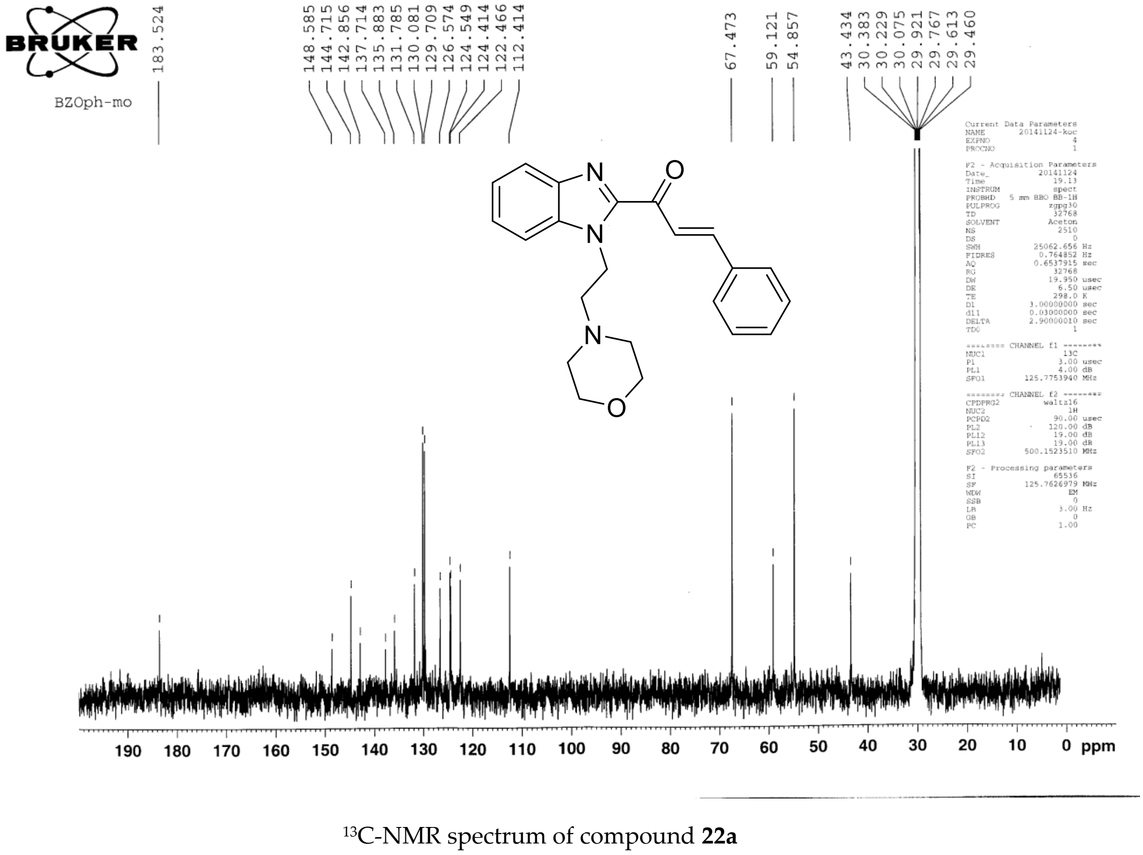

^{13}C-NMR spectrum of compound **22a**

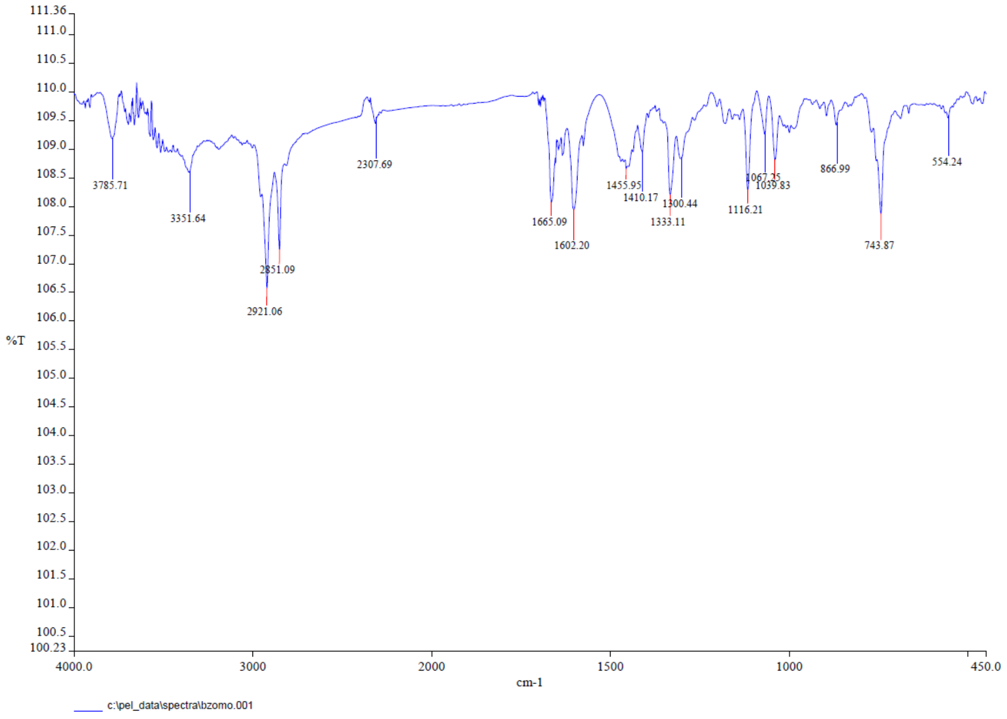

IR spectrum of compound **22a**

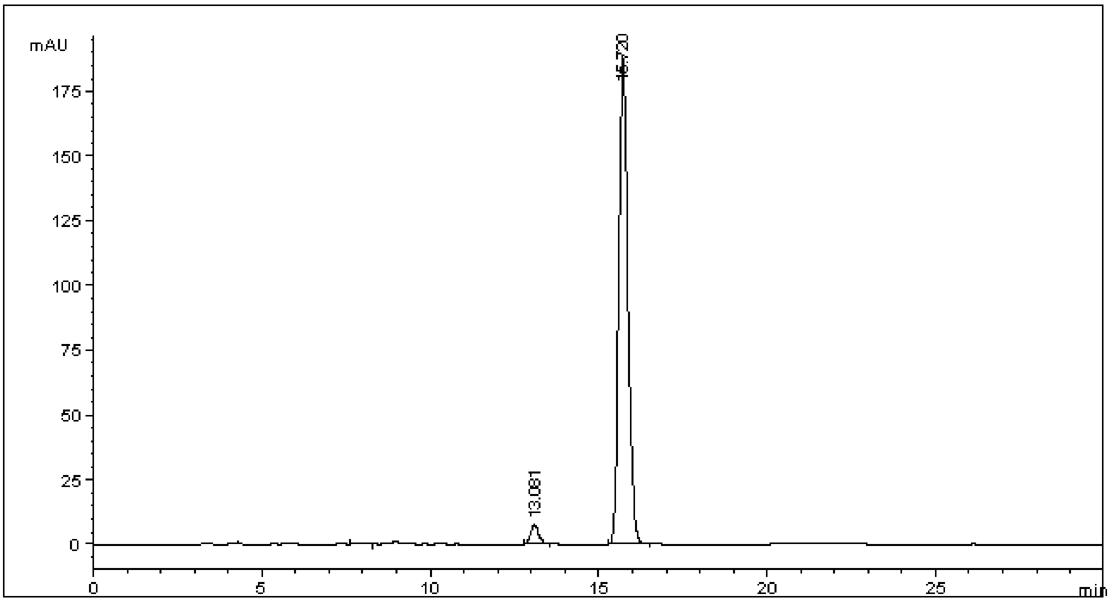

HPLC spectrum of compound **22a**

Peak #	RetTime [min]	Width [min]	Area mAU *s	Area %
1	13.081	0.263	131.5152	3.5487
2	15.72	0.2962	3574.548	96.4513

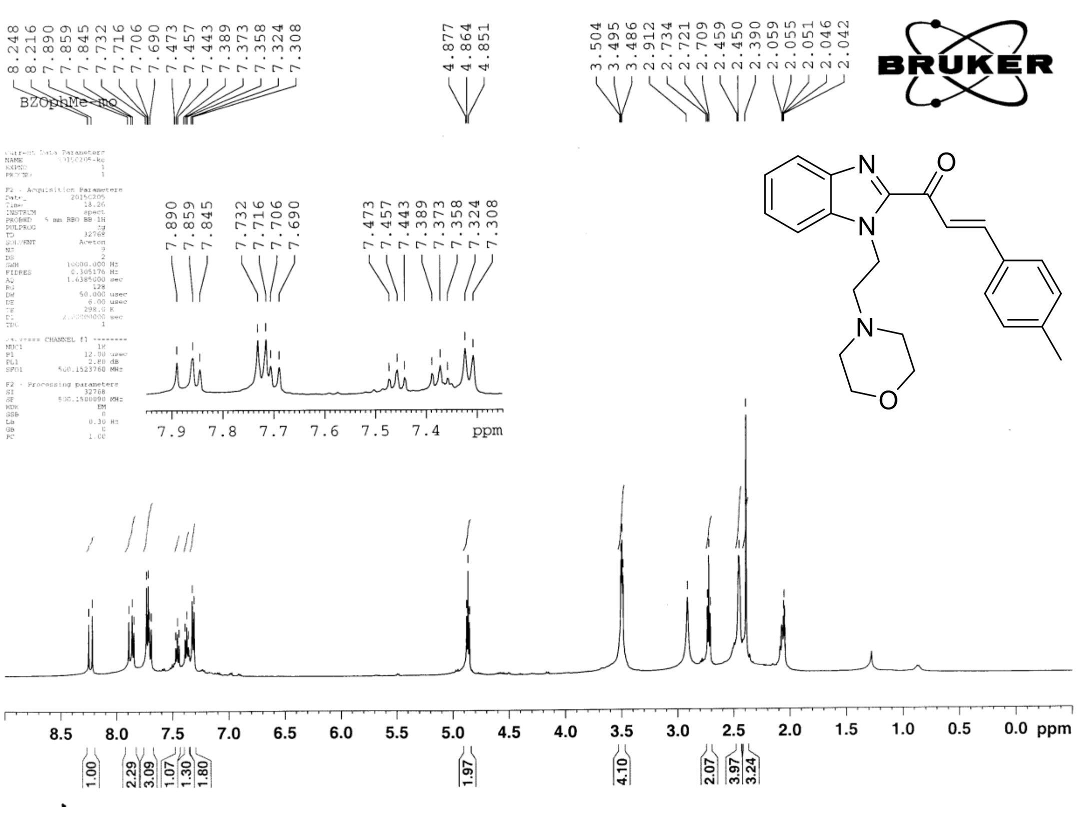

^{1}H-NMR spectrum of compound **22b**

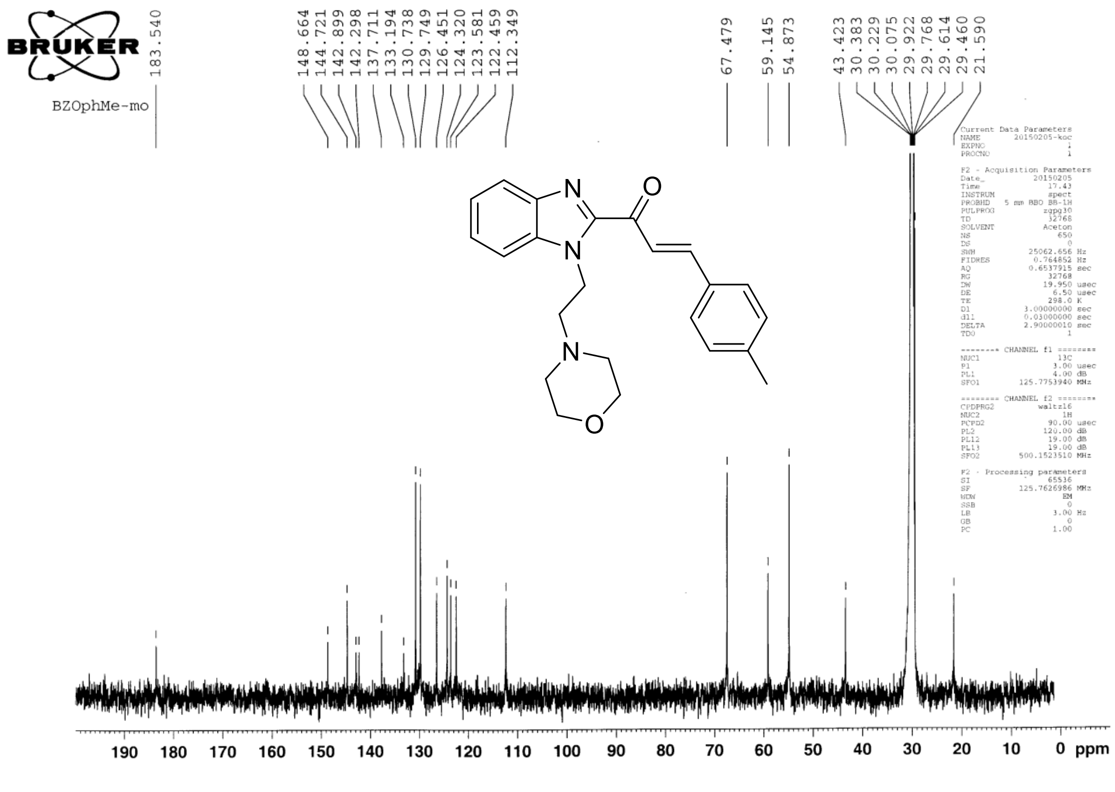

^{13}C-NMR spectrum of compound **22b**

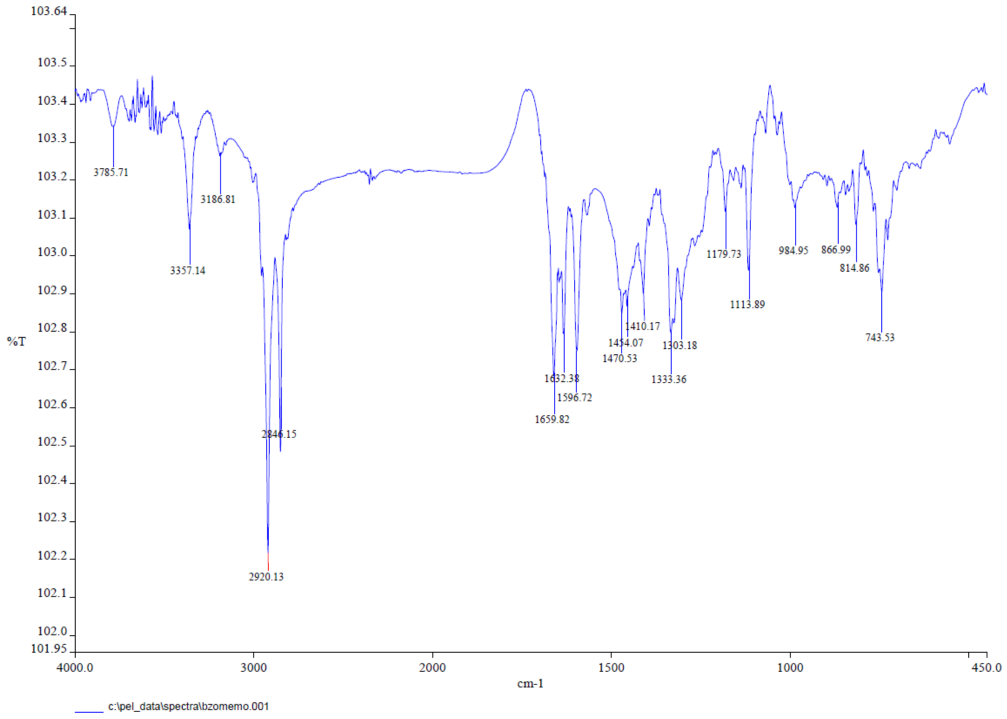

IR spectrum of compound **22b**

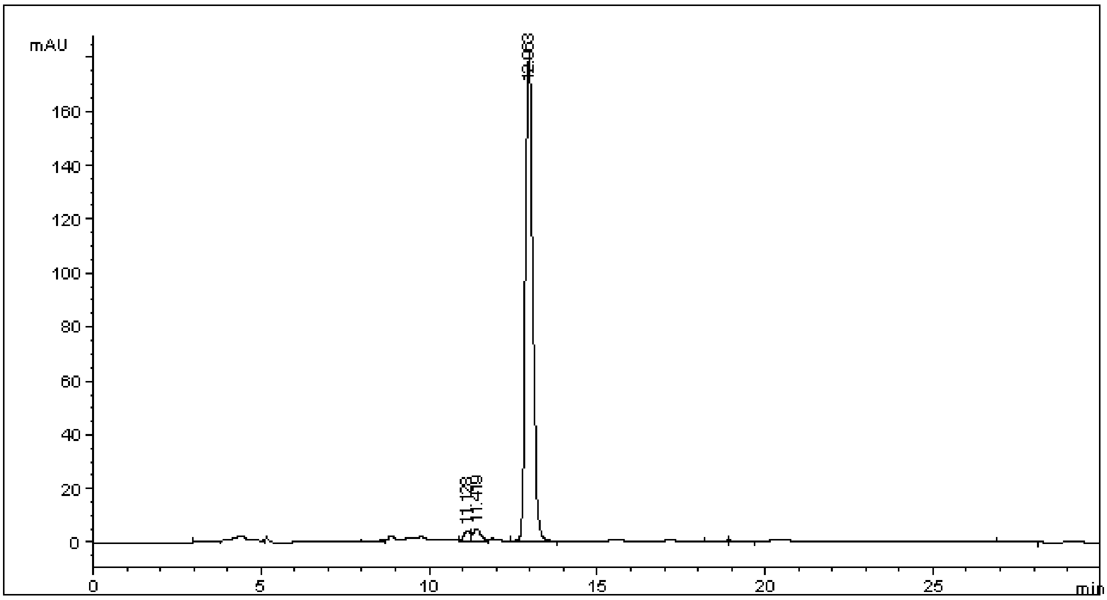

HPLC spectrum of compound **22b**

Peak #	RetTime [min]	Width [min]	Area mAU *s	Area %
1	11.128	0.2204	51.86035	1.8558
2	11.419	0.2363	68.21777	2.4412
3	12.963	0.2307	2674.29761	95.7029

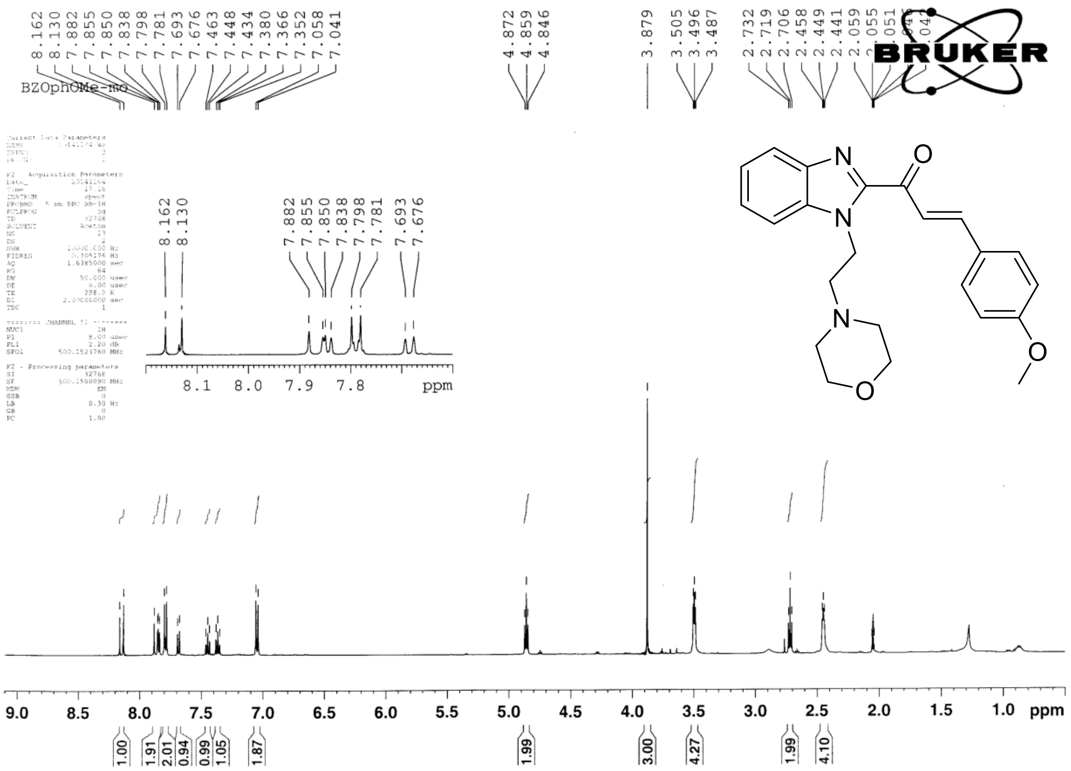

^{1}H-NMR spectrum of compound **22c**

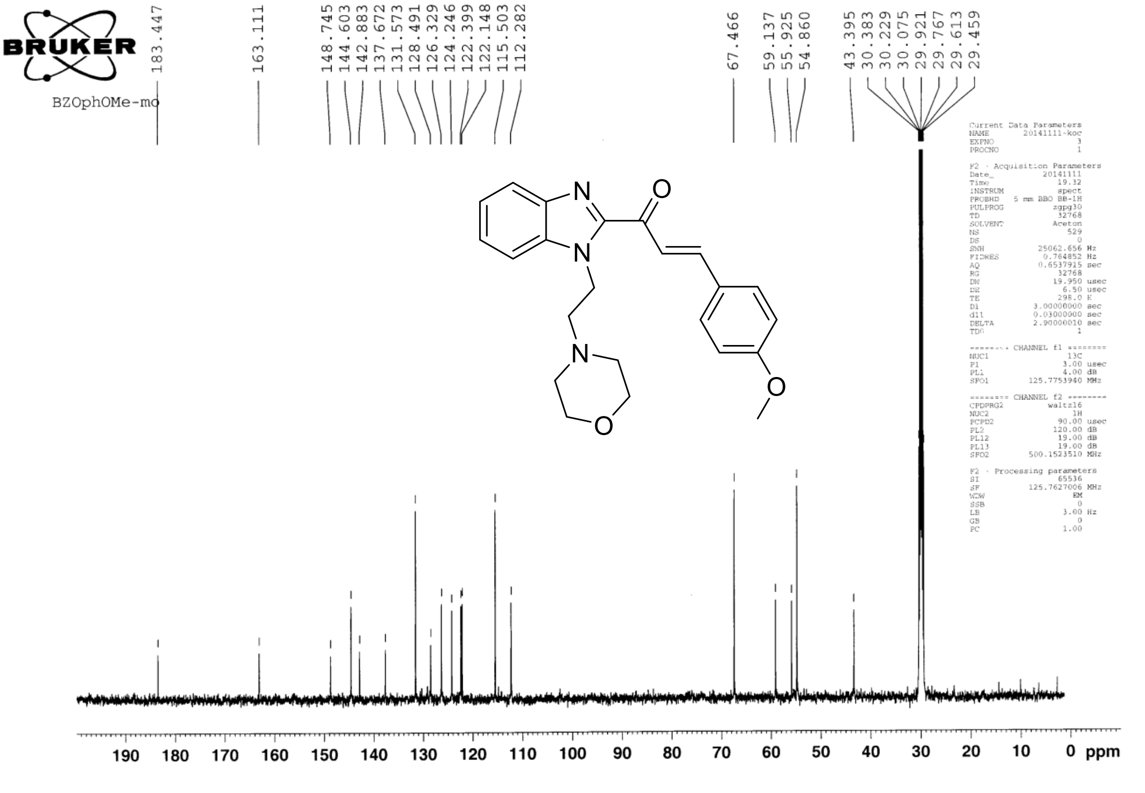

^{13}C-NMR spectrum of compound **22c**

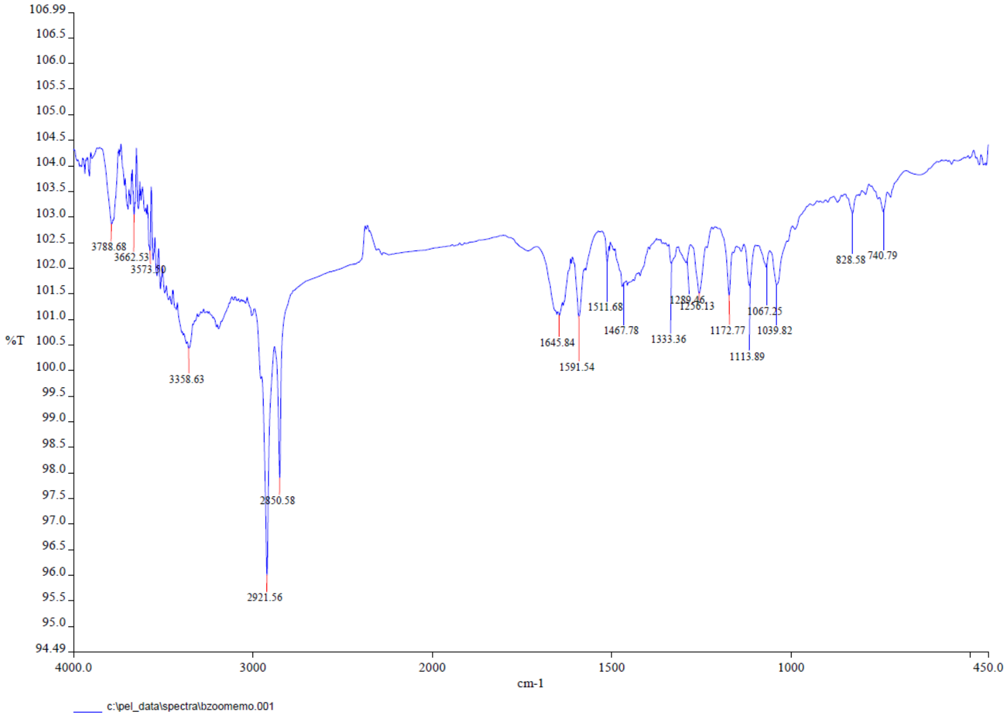

IR spectrum of compound **22c**

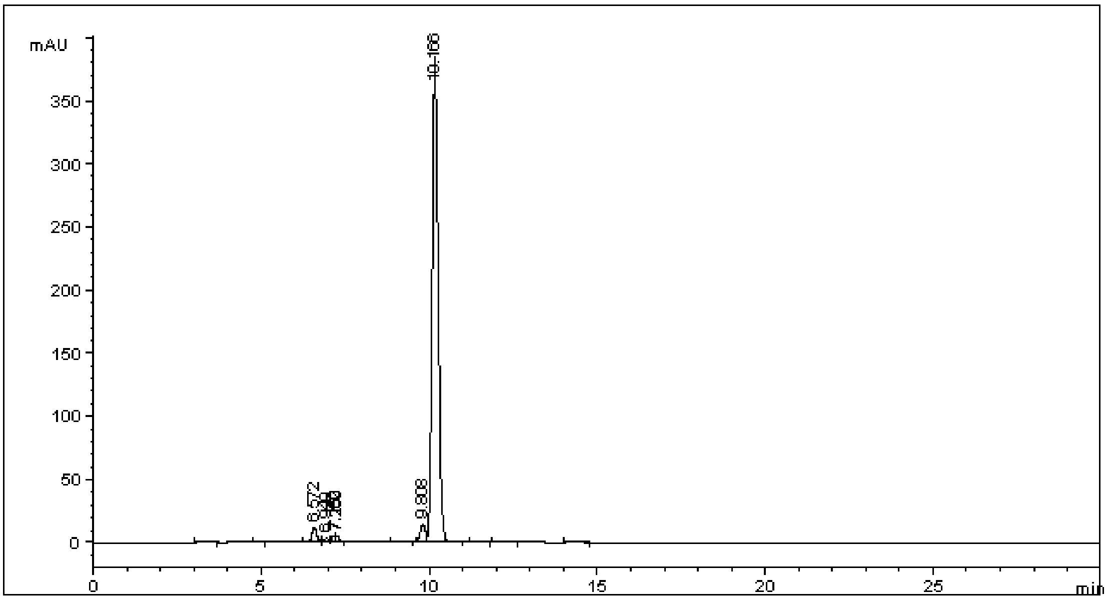

HPLC spectrum of compound **22c**

Peak #	RetTime [min]	Width [min]	Area mAU *s	Area %
1	6.949	0.1349	25.79159	0.5232
2	7.162	0.1196	32.28178	0.6549
3	7.25	0.1134	27.91112	0.5662
4	9.808	0.1719	153.38657	3.1118
5	10.166	0.1891	4689.81201	95.1438

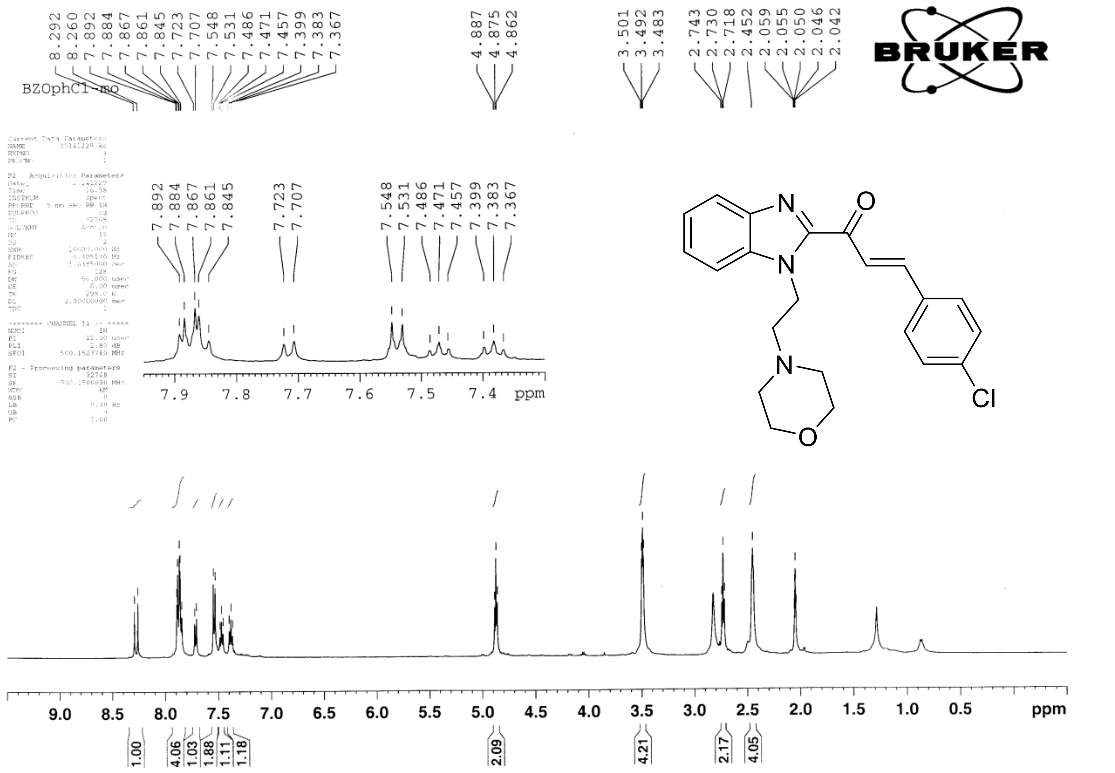

^{1}H-NMR spectrum of compound **22d**

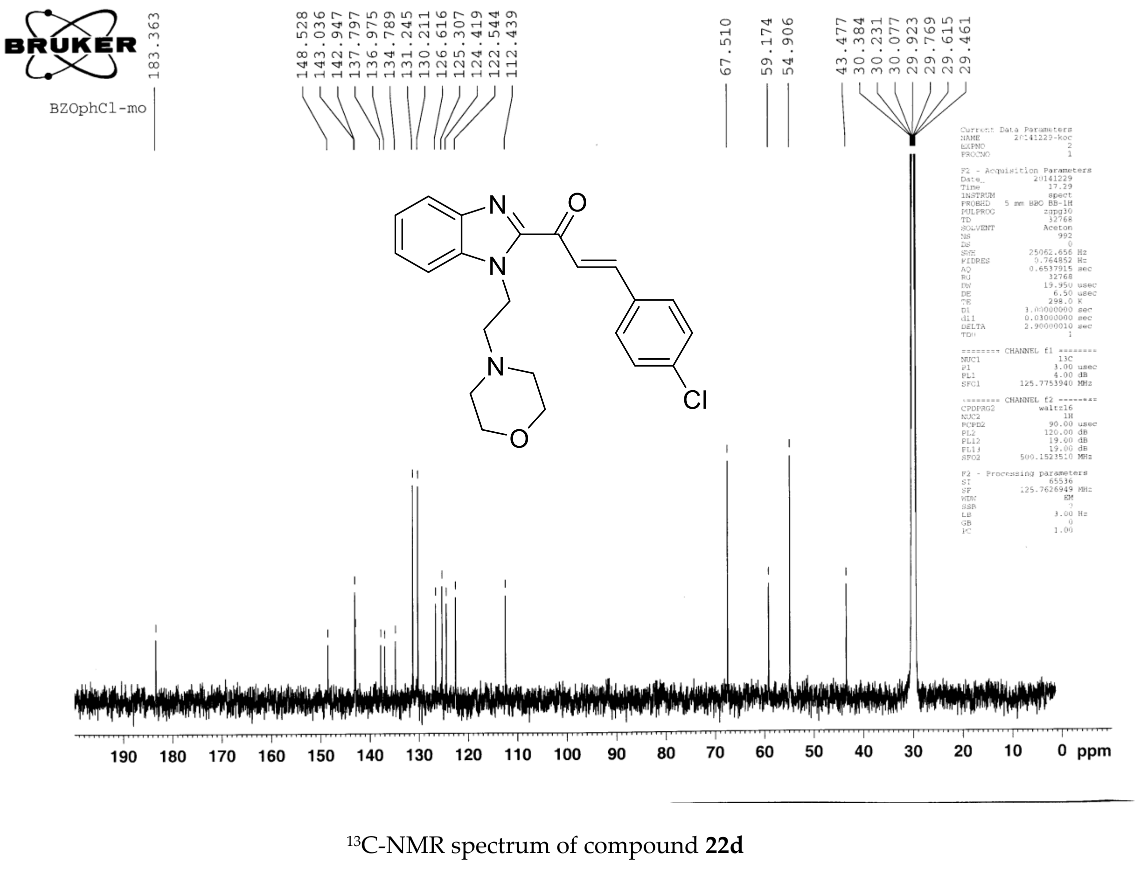

^{13}C-NMR spectrum of compound **22d**

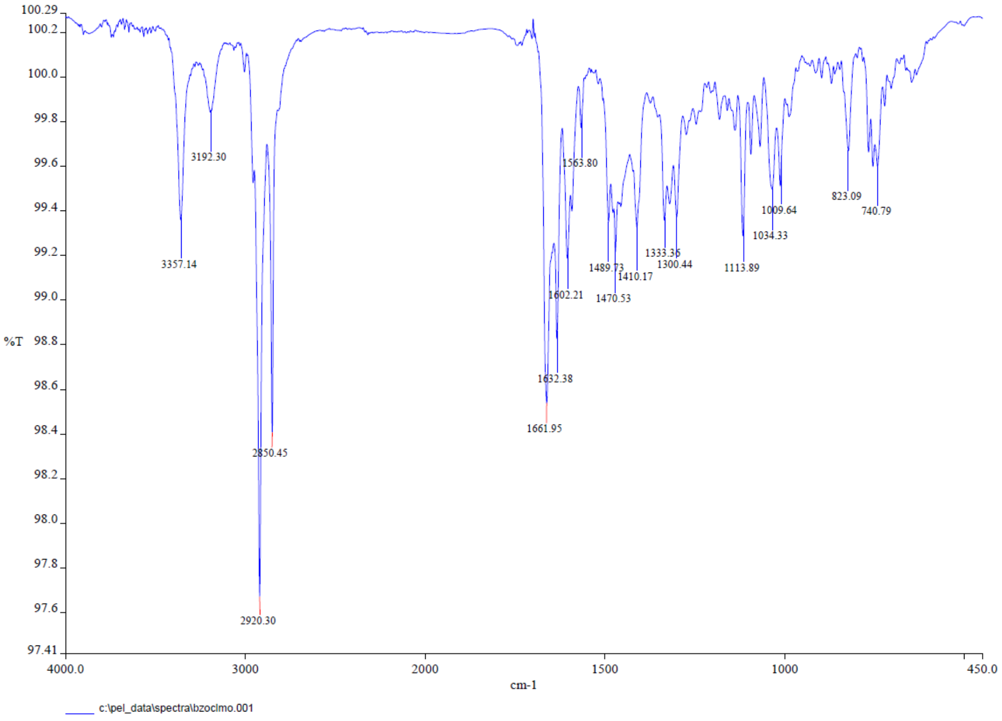

IR spectrum of compound **22d**

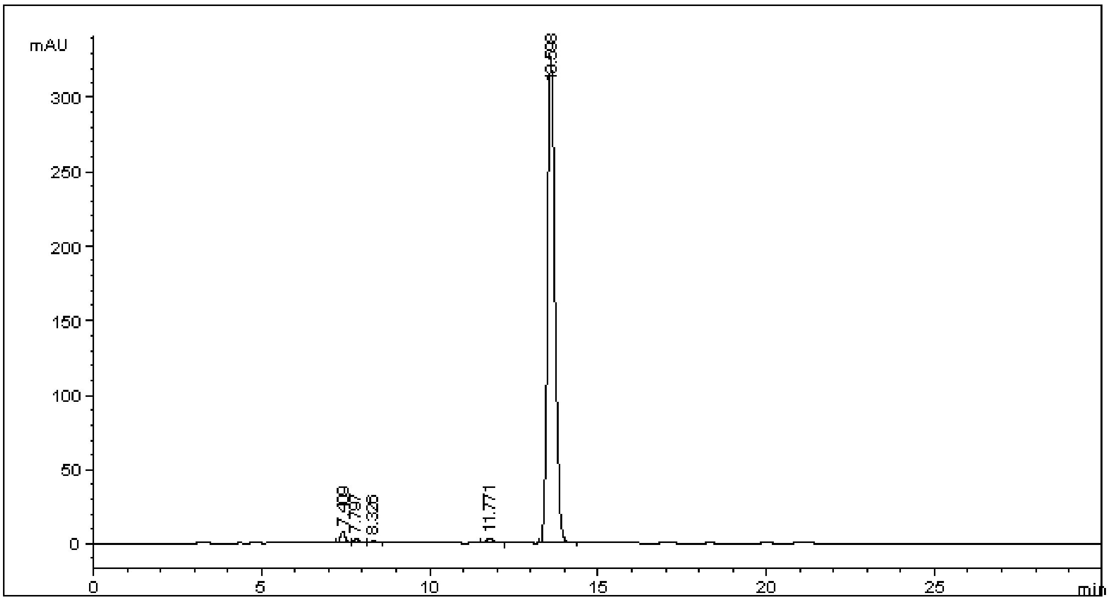

HPLC spectrum of compound **22d**

Peak #	RetTime [min]	Width [min]	Area mAU *s	Area %
1	7.409	0.1596	80.65369	1.5400
2	7.797	0.1728	40.02195	0.7642
3	8.326	0.1800	26.33945	0.5029
4	11.771	0.2311	50.84013	0.9708
5	13.598	0.2397	5039.33447	96.2221

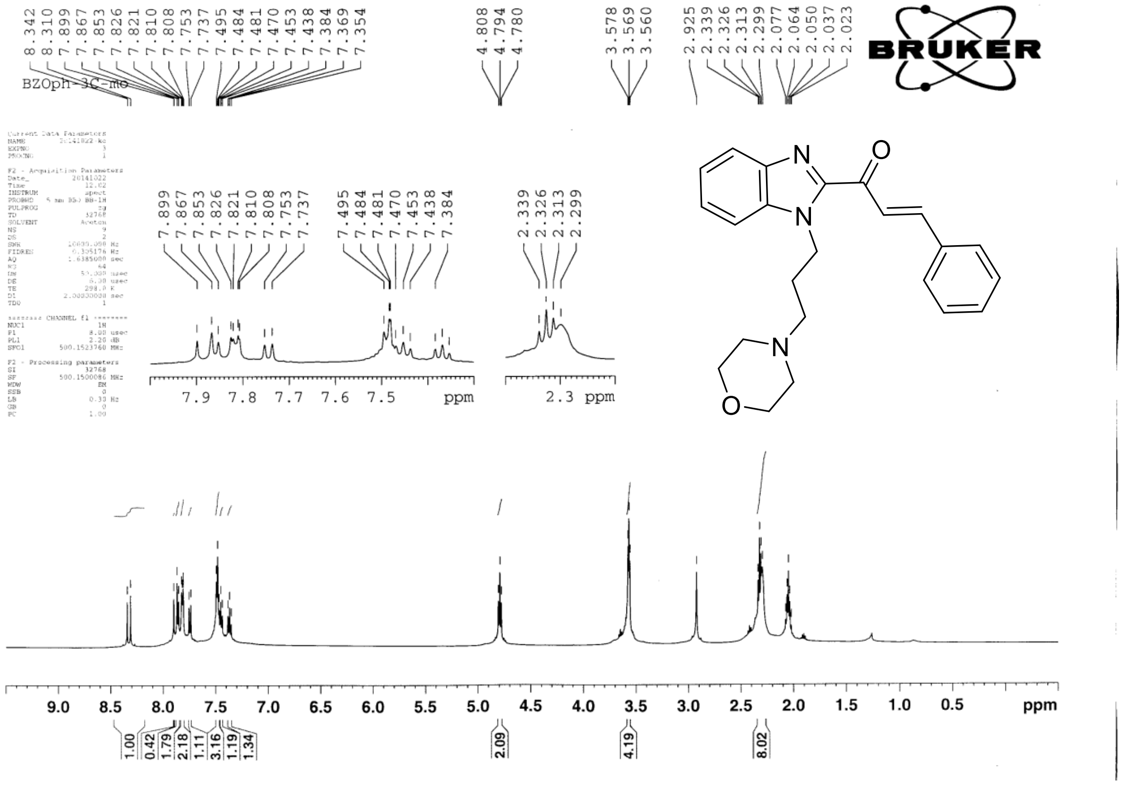

^{1}H-NMR spectrum of compound **23a**

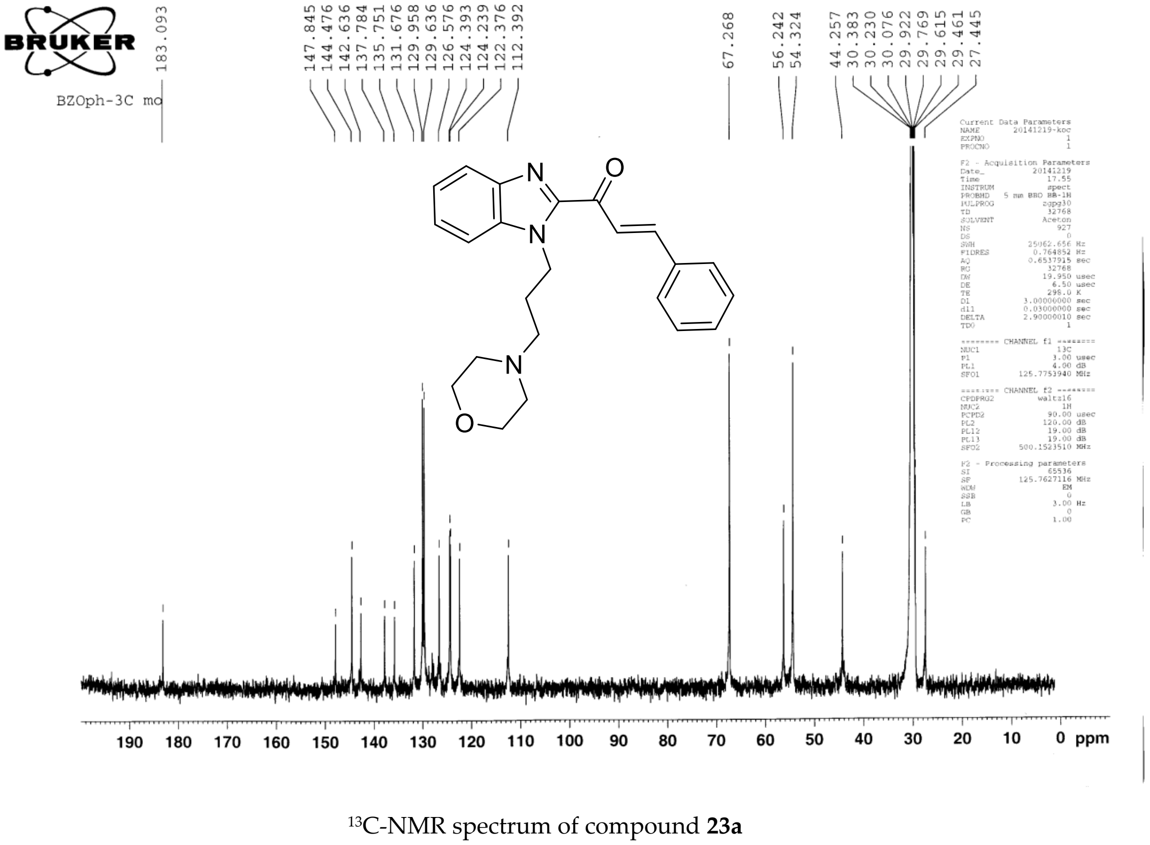

^{13}C-NMR spectrum of compound **23a**

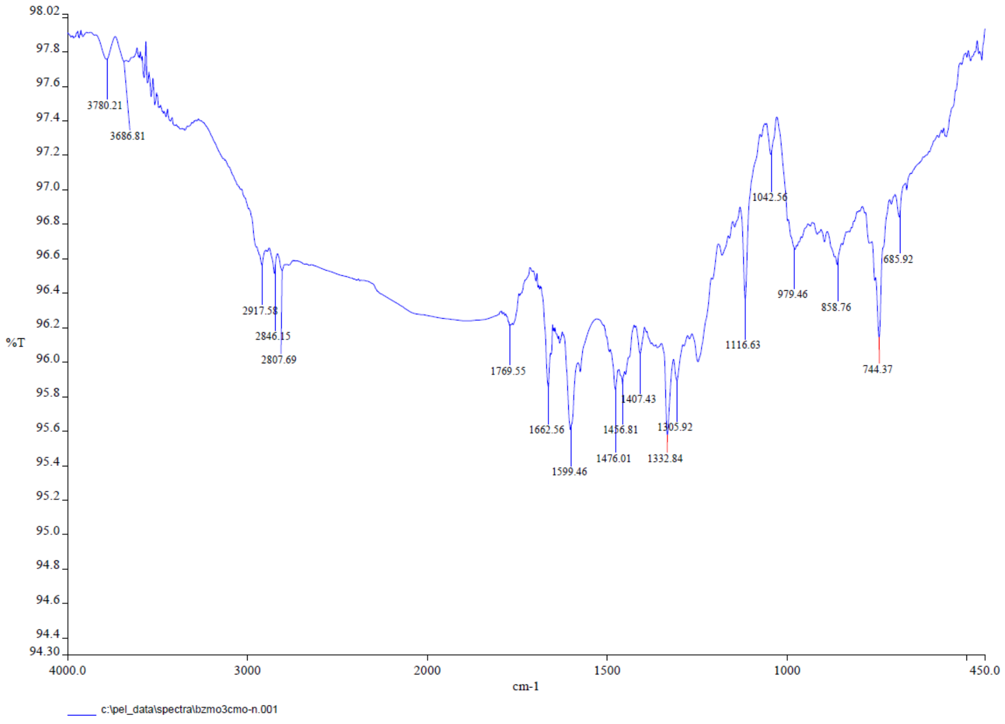

IR spectrum of compound **23a**

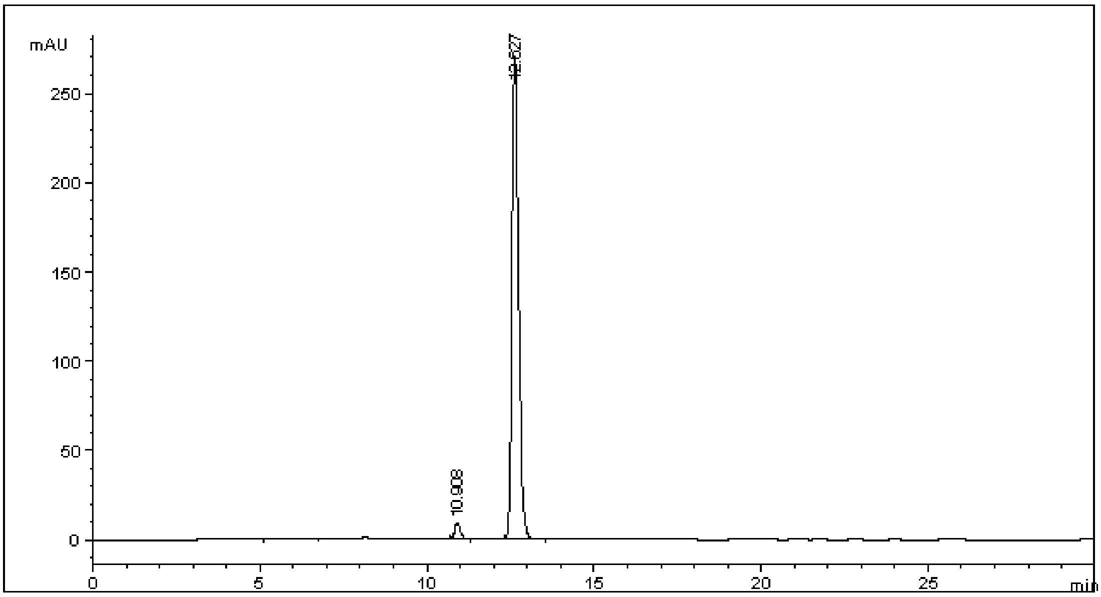

HPLC spectrum of compound **23a**

Peak #	RetTime [min]	Width [min]	Area mAU *s	Area %
1	10.908	0.1868	105.05498	2.6781
2	12.627	0.2189	3817.72192	97.3219

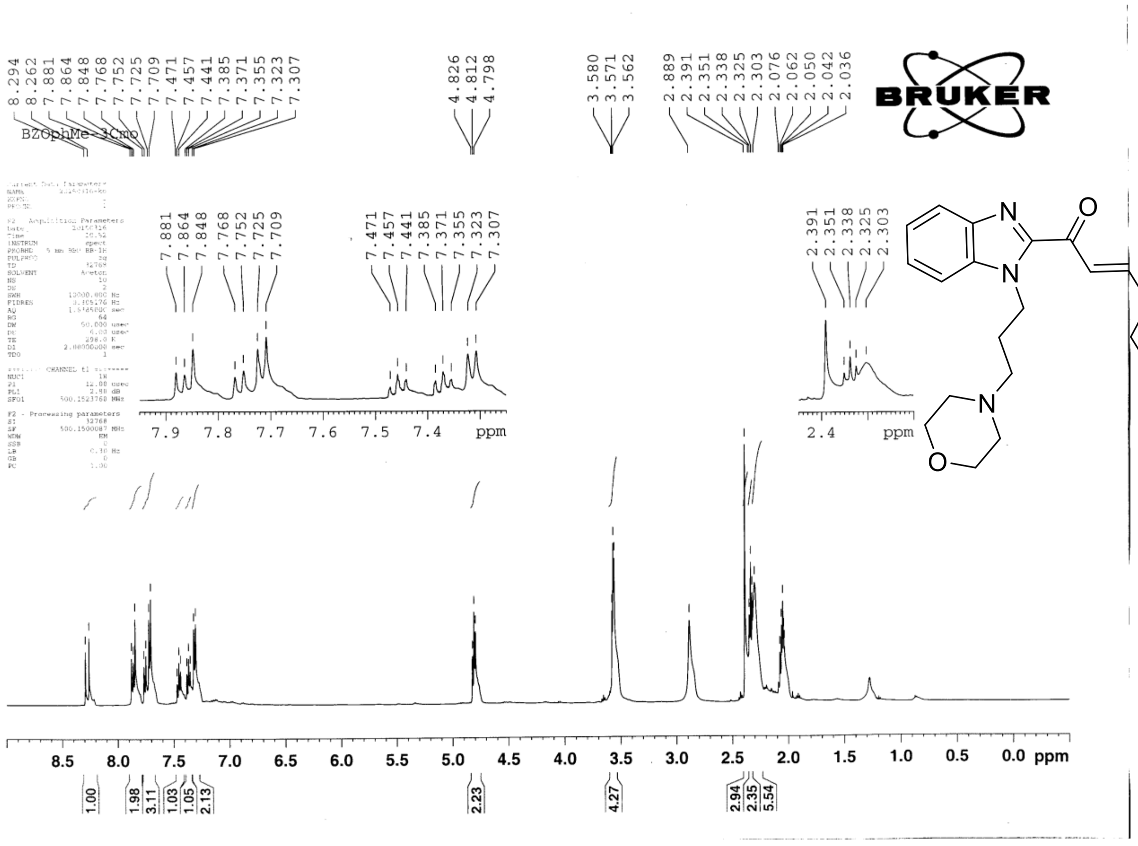

^{1}H-NMR spectrum of compound **23b**

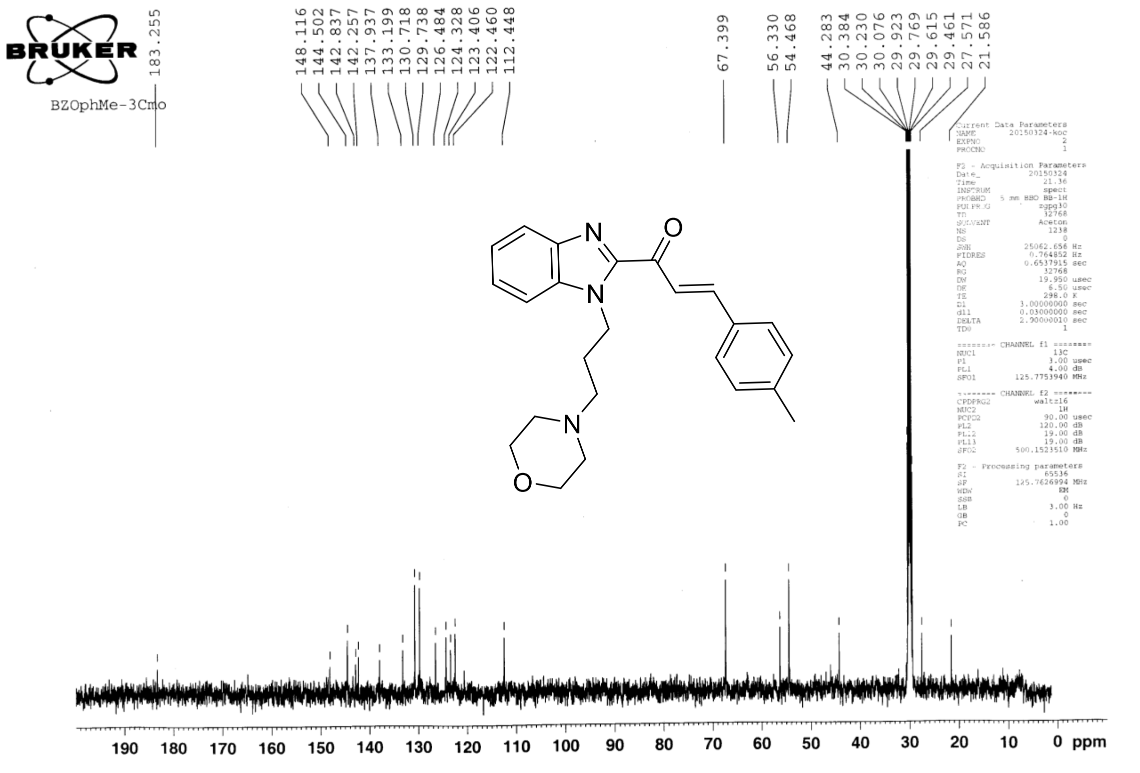

^{13}C-NMR spectrum of compound **23b**

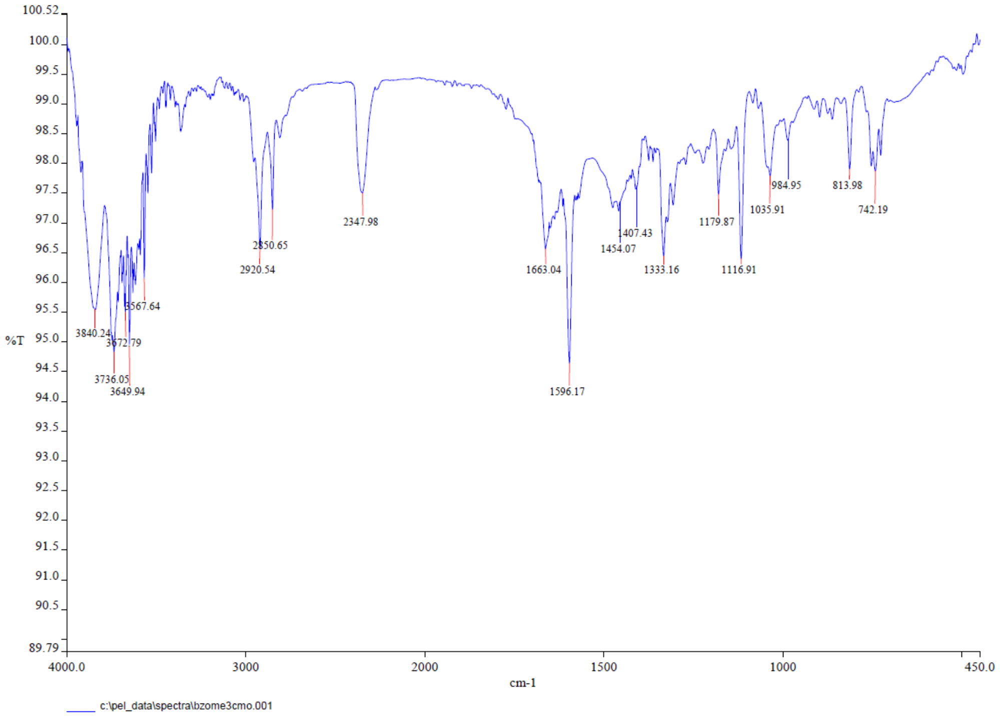

IR spectrum of compound **23b**

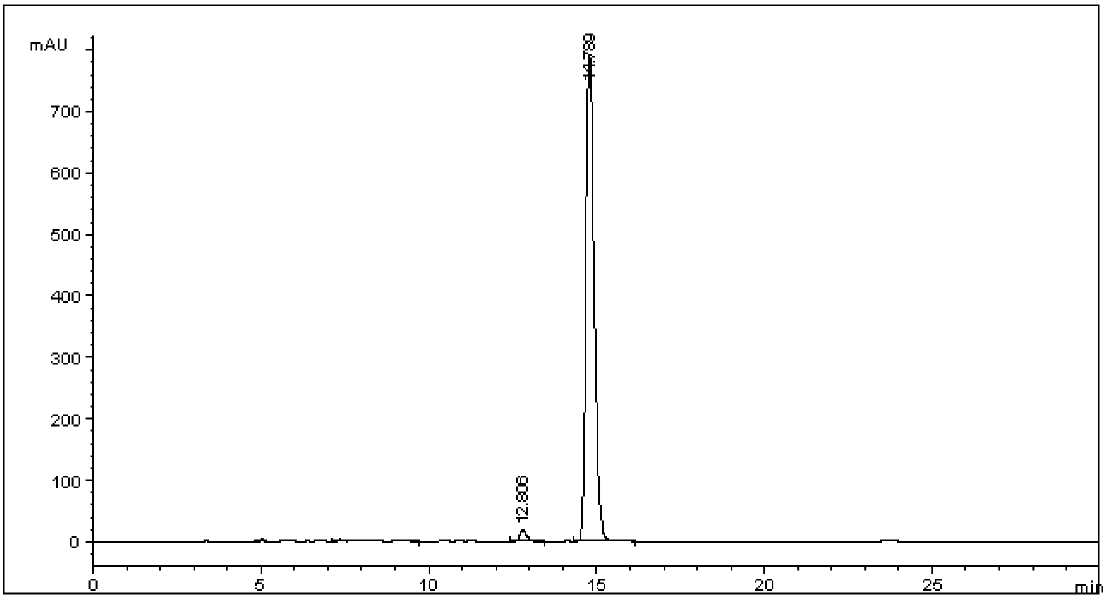

HPLC spectrum of compound **23b**

Peak #	RetTime [min]	Width [min]	Area mAU *s	Area %
1	12.806	0.2203	278.77322	2.0574
2	14.789	0.2576	13271.3	97.9426

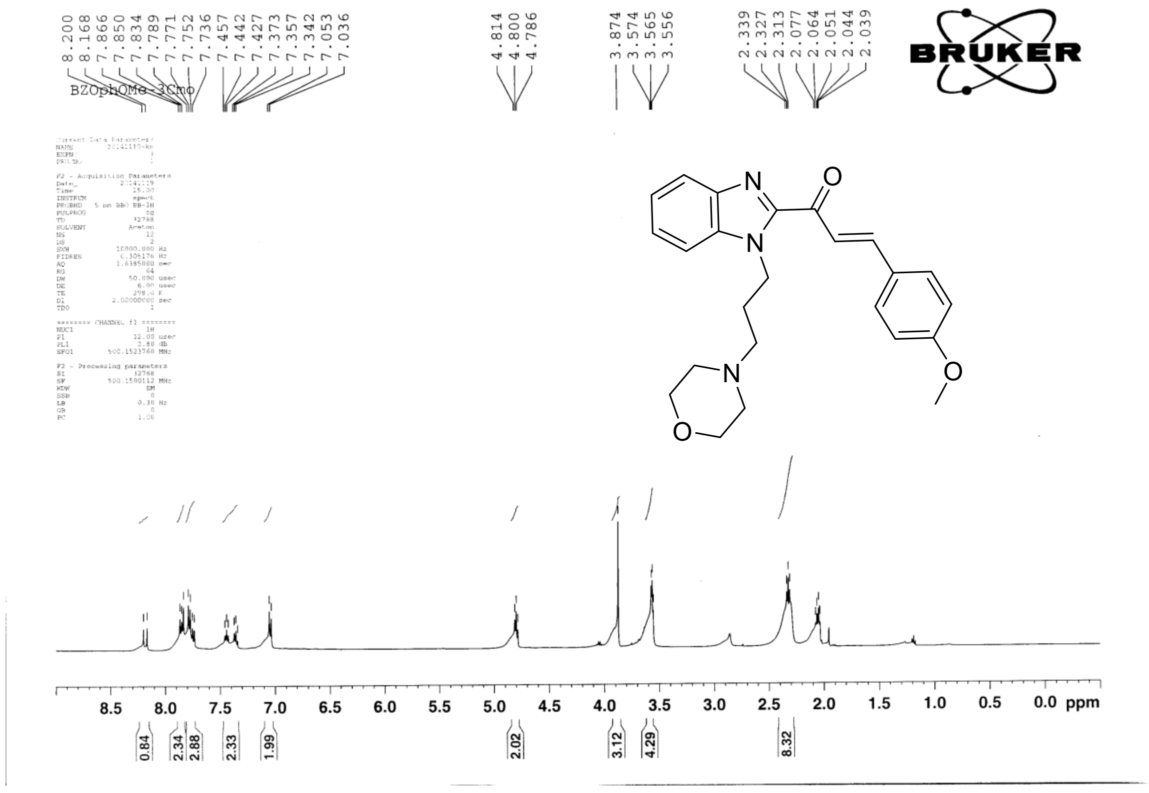

^{1}H-NMR spectrum of compound **23c**

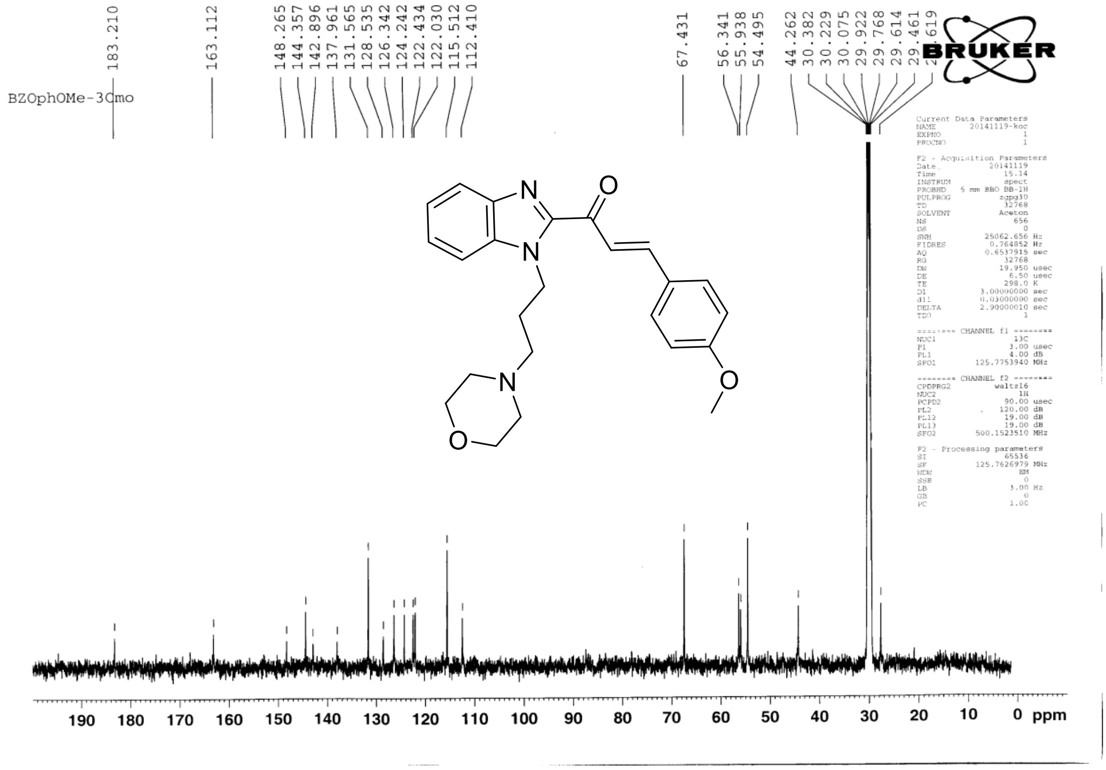

^{13}C-NMR spectrum of compound **23c**

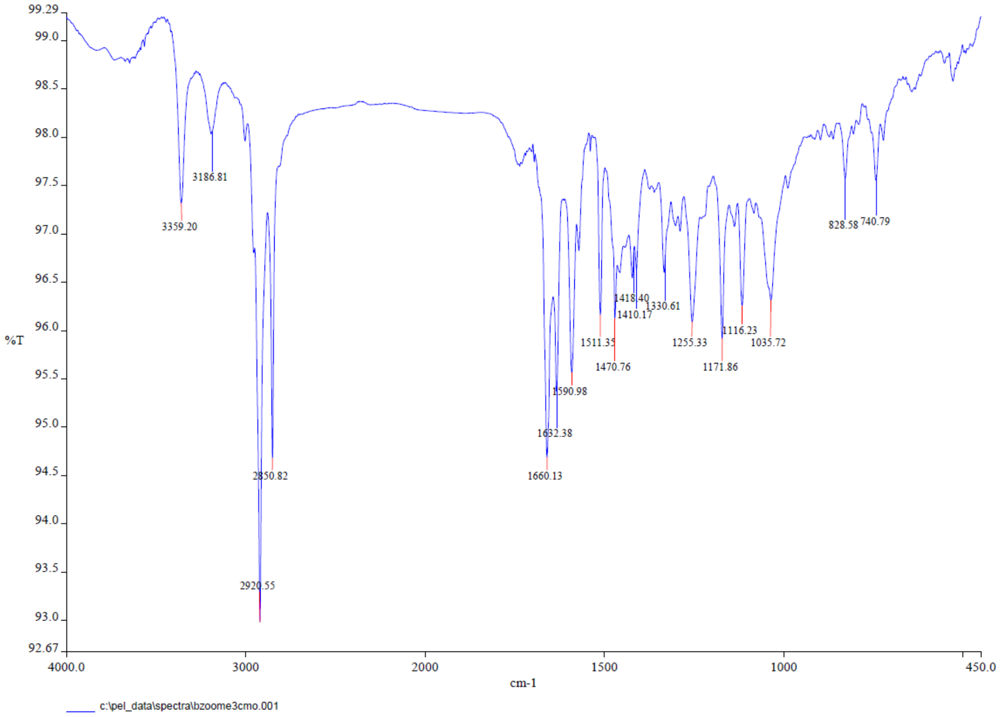

IR spectrum of compound **23c**

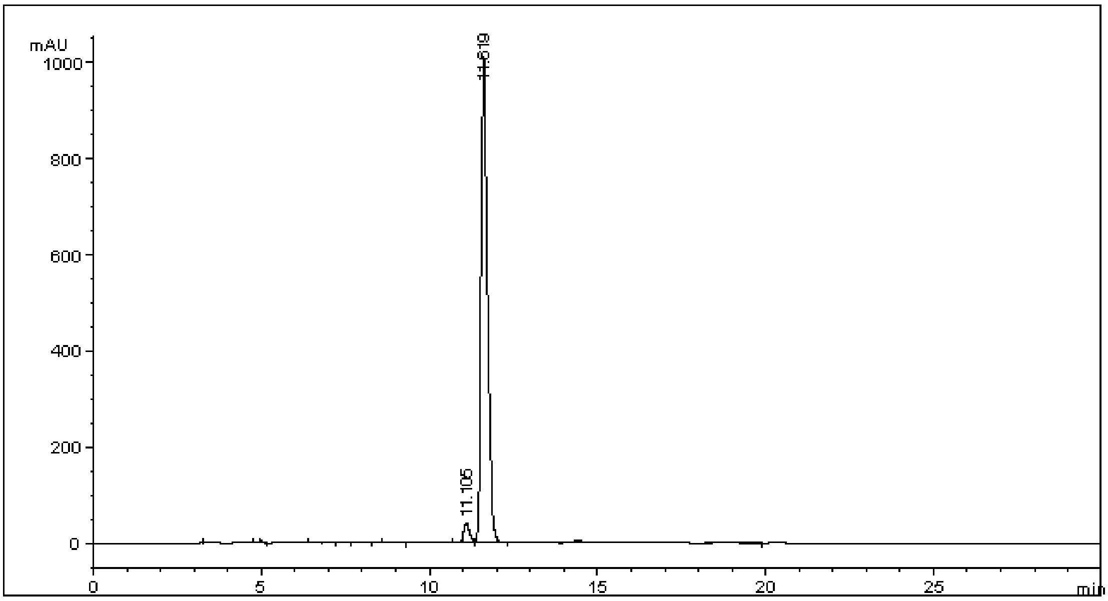

HPLC spectrum of compound **23c**

Peak #	RetTime [min]	Width [min]	Area mAU *s	Area %
1	11.105	0.1937	513.34729	3.631
2	11.619	0.2083	13623.2	96.369

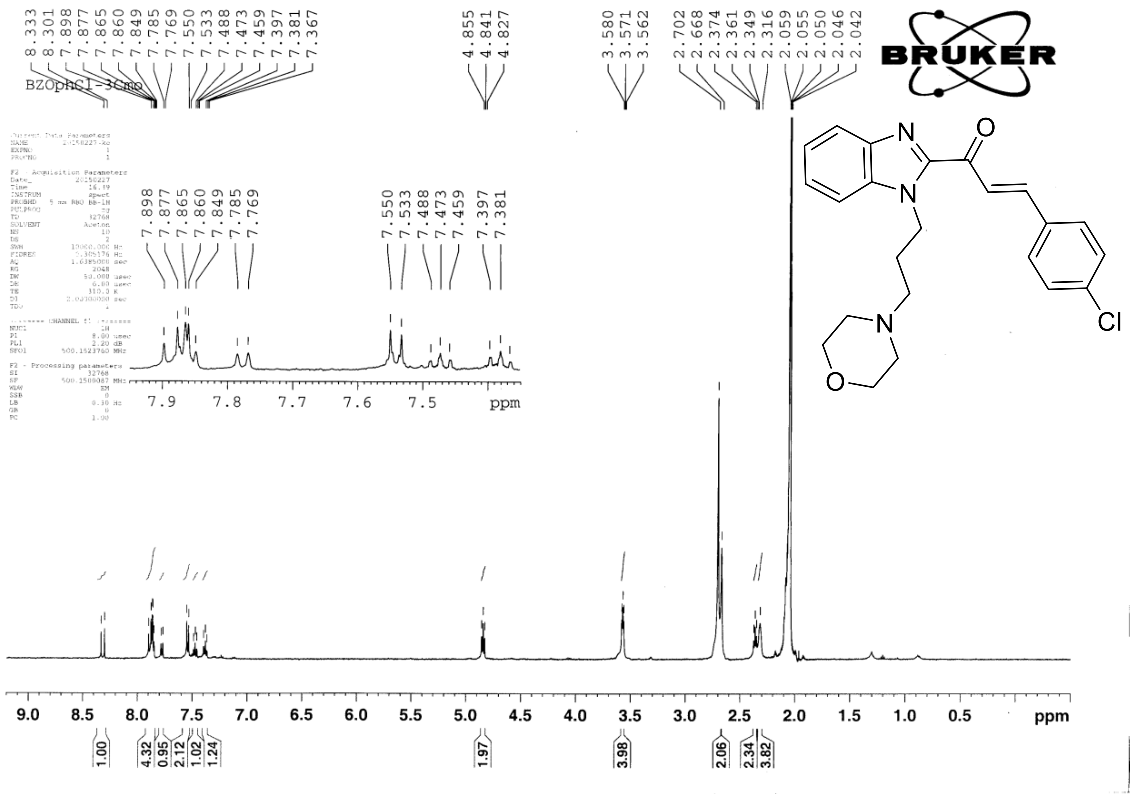

^{1}H-NMR spectrum of compound **23d**

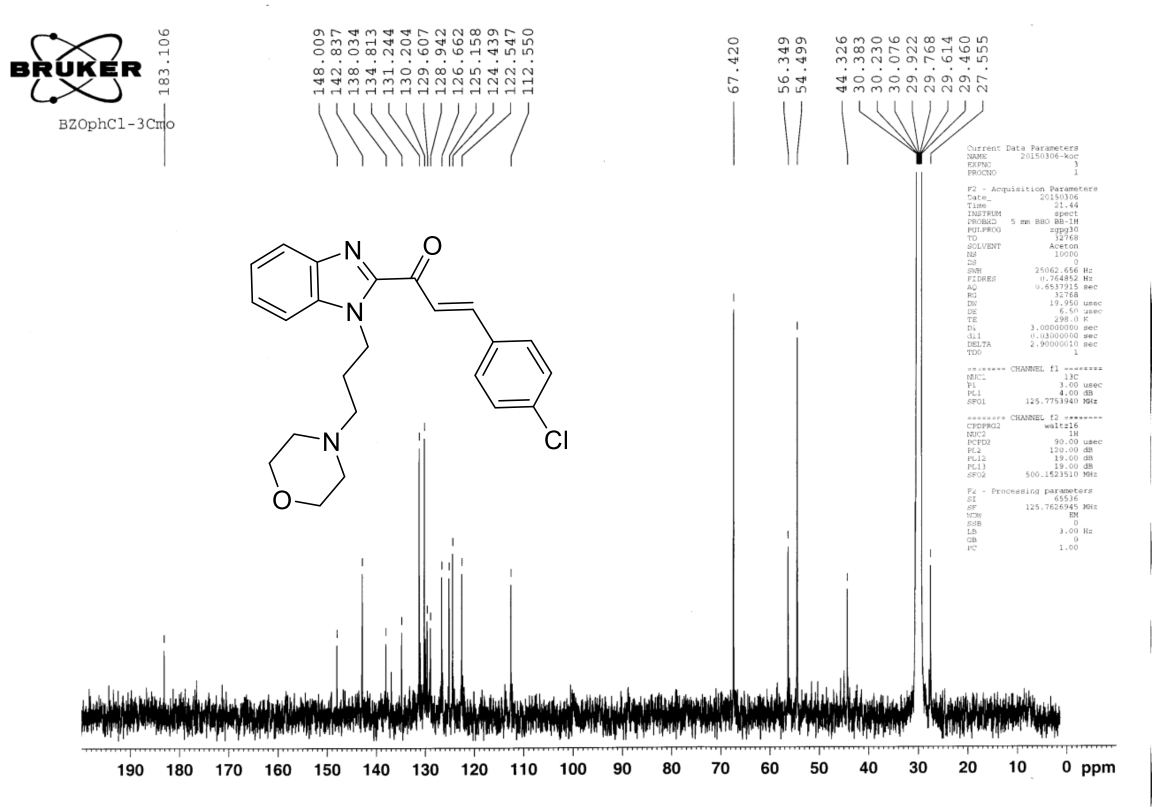

^{13}C-NMR spectrum of compound **23d**

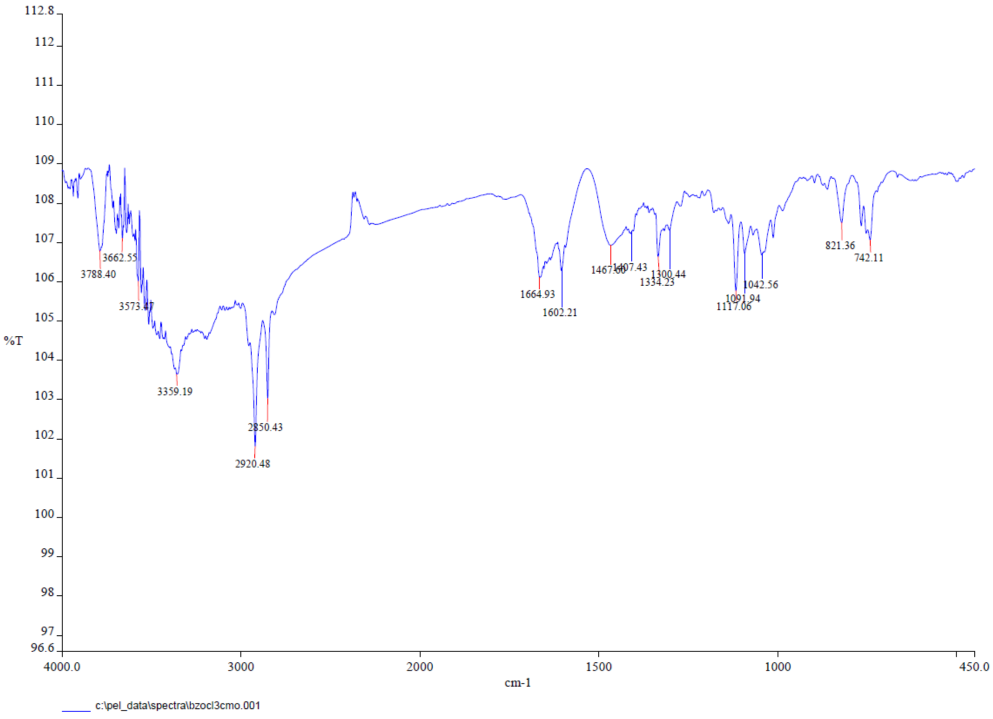

IR spectrum of compound **23d**

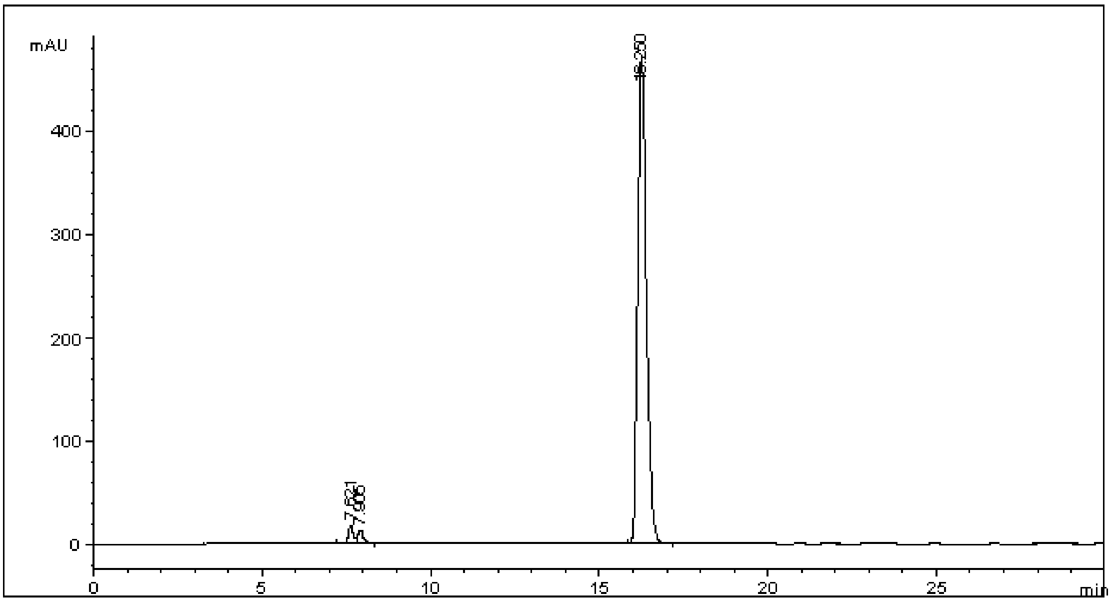

HPLC spectrum of compound **23d**

Peak #	RetTime [min]	Width [min]	Area mAU *s	Area %
1	7.621	0.1547	187.74409	2.1252
2	7.905	0.1755	161.00226	1.8225
3	16.250	0.2775	8485.20020	96.052

(2) Flow Cytometry Data

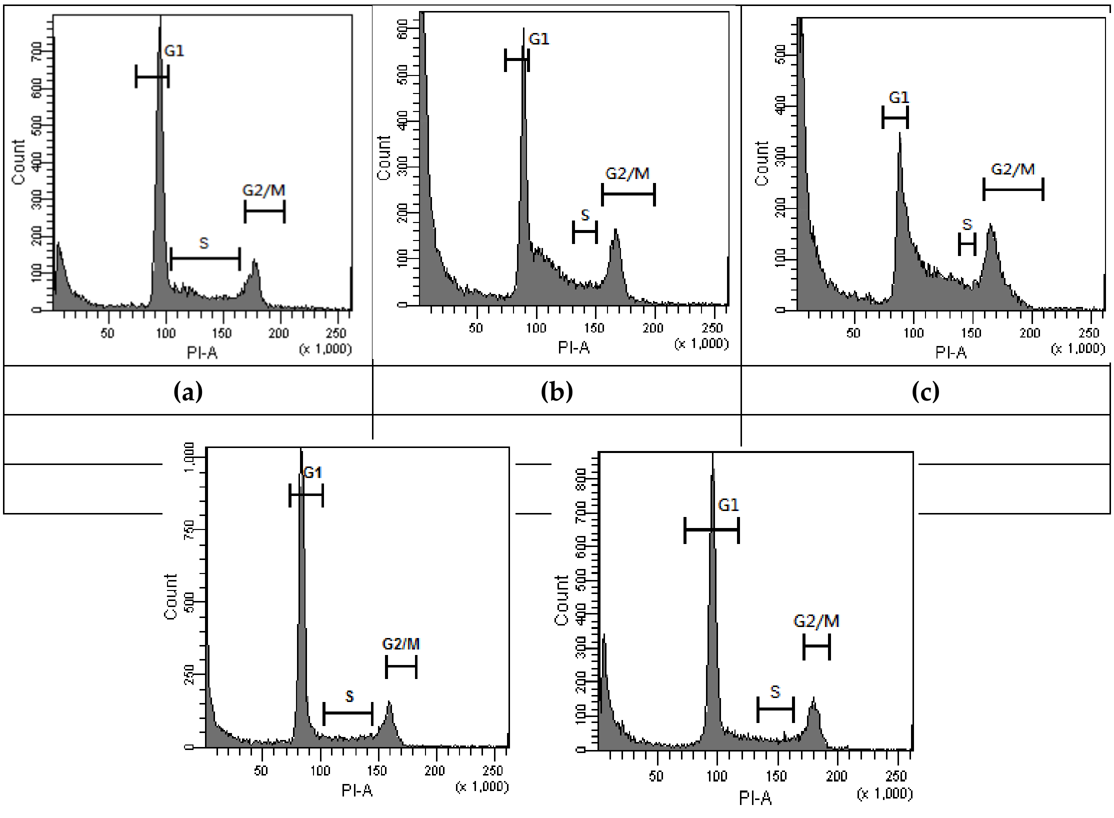

(d) **(e)**

Figure S1. DNA histograms of exponentially growing OVCAR-3 cells with different treatments: (a) cell only; (b) compound **20a**; (c) compound **21a**; (d) compound **22a**; (e) compound **23a**.
